# Bioeliminable Pt@Bi_2_Se_3_-RGD Nanoassembly for Enhancing Photoacoustic Imaging-Guided Tumor Immuno-Radiotherapy by Inducing Apoptosis via the Areg Pathway

**DOI:** 10.7150/thno.106479

**Published:** 2025-01-27

**Authors:** Huanhuan Tan, Shiyan Fu, Li Shen, Qinyang Lin, Wenrun Li, Yonghong Ran, Yazhen Zhao, Longfei Tan, Yuhui Hao

**Affiliations:** 1 State Key Laboratory of Trauma and Chemical Poisoning, Institute of Combined Injury, Chongqing Engineering Research Center for Nanomedicine, College of Preventive Medicine, Army Medical University, Chongqing 400038, China.; 2 Laboratory of Controllable Preparation and Application of Nanomaterials, Key Laboratory of Cryogenics, Technical Institute of Physics and Chemistry, Chinese Academy of Sciences, 29 Zhongguancun East Road, Beijing 100190, China.

**Keywords:** Pt@Bi_2_Se_3_-RGD, radiosensitization, hypoxia, immuno-radiotherapy, bioeliminable

## Abstract

**Background:** Nanoradiosensitizers containing high Z-group elements have been reported widely as potential candidates for radiotherapy. However, the specific regulatory mechanism is unclear, and biodegradability needs to be addressed urgently.

**Methods:** We synthesized a silk sericin-containing nano assembly, Pt@Bi_2_Se_3_-RGD (PBR). PBR's antitumor and bioeliminable effects were demonstrated in 4T1 tumor cells *in vitro* and *in vivo*. The immuno-radiotherapy effects of PBR were evaluated using a bilateral tumor model.

**Results:** Combining photoacoustic imaging-guided PBR with radiotherapy improved the efficiency of anti-PD-L1 treatment, eliciting a robust immune response. Importantly, silk sericin-containing PBR could respond to the local intracellular environment in the tumor with acidic pH and overexpressed MMP-9, collapsing into Bi, Se, and scattered Pt nanoparticles (NPs) and finally be cleared from the body. The results also suggested that PBR may act on the Areg/Egfr/Bcl-2 pathway, inducing apoptosis for radiosensitization.

**Conclusion:** The multifunctional, bioeliminable PBR nanoassembly synthesized in this study demonstrated radiosensitization, which, in conjunction with the PD-L1 immune blockade, could suppress primary and distal tumors. Thus, as a sensitizer for synergistic radiotherapy and immunotherapy, PBR could have wide-ranging clinical applications in oncology.

## Introduction

Radiotherapy (RT) is a type of local treatment that has been extensively used to treat most patients with breast cancer at different stages 1-3. RT uses high-energy radiation to directly ionize DNA molecules or indirectly interact with water molecules, forming reactive oxygen species (ROS) to induce cell apoptosis, ferroptosis, and necrosis 4-7. However, the immunosuppressive tumor microenvironment (TME) and damage to surrounding normal tissues by ionizing radiation limit its efficiency significantly 8-10. Radiosensitizers currently used in the clinic are small molecule drugs with a quick metabolism, low targeting efficiency, and significant toxic side effects, making achieving the ideal therapeutic effect difficult 11-12. Nanoradiosensitizers with a high atomic number (Au, Hf, W, and Bi) have been reported to improve the efficacy of RT by promoting intracellular radiation energy deposition and triggering robust cancer immunotherapy 13-18. HfO_2_ nanoparticles NBTXR3 showed encouraging radiological and pathologic responses in patients with soft tissue sarcoma in clinical trials 19,20. Hypoxic TME also restricts the therapeutic efficiency of RT 21,22. Several studies have reported oxygen delivery by red blood cells or oxygenated perfluorocarbon 23,24. However, oxygen delivery and release efficiency were unsatisfactory 25. Pt nanozymes have successfully been used to ameliorate hypoxia to promote cancer treatment 26-28. Therefore, designing and exploring nanozymes with high Z-group elements to overcome hypoxia is a promising approach to realizing RT sensitization.

Although nanoradiosensitizers containing high Z-group elements are potential candidates for RT, few studies on bioeliminable nanoradiosensitizers have been reported excepted the hollow silica and organic nanoparticles 29-31. Introducing proteins into the preparation process of inorganic nanomaterials seems promising 32,33. Sericin is often used to prepare biomaterials because of its good biocompatibility and richness of amino and carboxyl groups, which are beneficial for modification 34,35. Matrix metalloproteinases (MMPs), such as MMP-9, routinely overexpressed in most human cancers, can also decompose peptides obtained from collagen 36-38.

**Figure 1:**
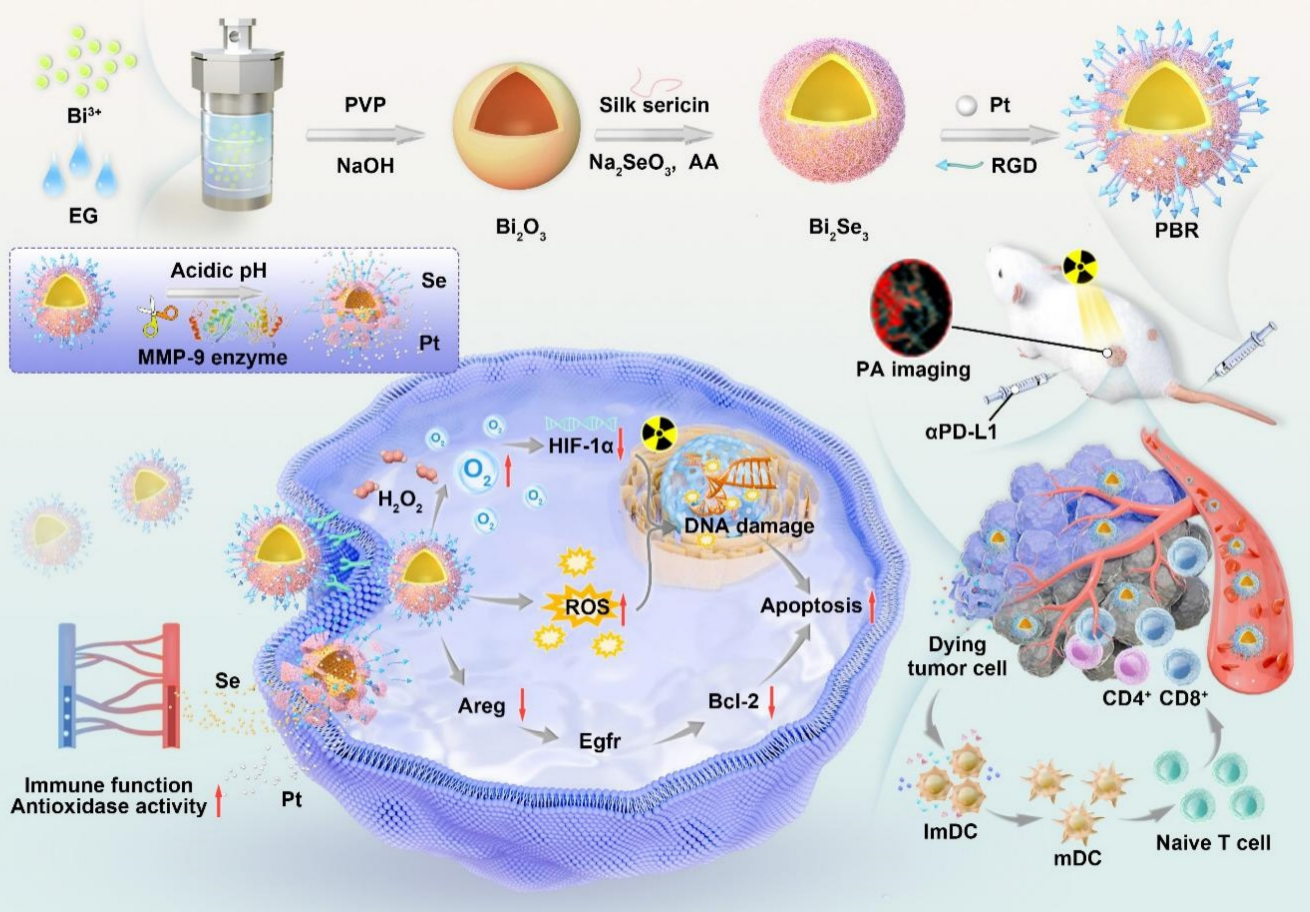
Synthesis of bioeliminable PBR and its proposed mechanism for mediating photoacoustic imaging-guided radioimmunotherapy.

Herein, we designed bioeliminable bismuth selenide nanoassembly Pt@Bi_2_Se_3_-RGD (PBR) for radiosensitization (**Figure 1**). Bi nanomaterials have shown considerable potential in effectively enhancing radial irradiation 39. Se, which plays a crucial role in maintaining human health as an essential trace element, can enhance semiconductor nanomaterials' catalytic activity and potentiate immune cells to induce robust antitumor immunity, combating tumor progression 40-43. The introduction of sericin protein in the synthesis of PBR can respond to the weak acidity of the tumor microenvironment and metal matrix proteases (MMP-9), leading to PBR accumulation in mice and decomposing into ions and discrete nanoparticles, which are ultimately cleared from the body through renal clearance, effectively addressing their biosafety issues. Catalase activity is attributed to platinum nanoparticles that can catalyze oxygen production from hydrogen peroxide in the tumor microenvironment, further improving the hypoxic microenvironment and promoting radiotherapy sensitization. The combination of radiotherapy sensitization mediated by PBR, the multifunctional bioeliminable nanoradiosensitizers synthesized in this study, and PD-L1 effectively amplifies the efficacy of radiotherapy and immunotherapy.

## Results and Discussion

### Structural characterization of PBR

**Figure 2:**
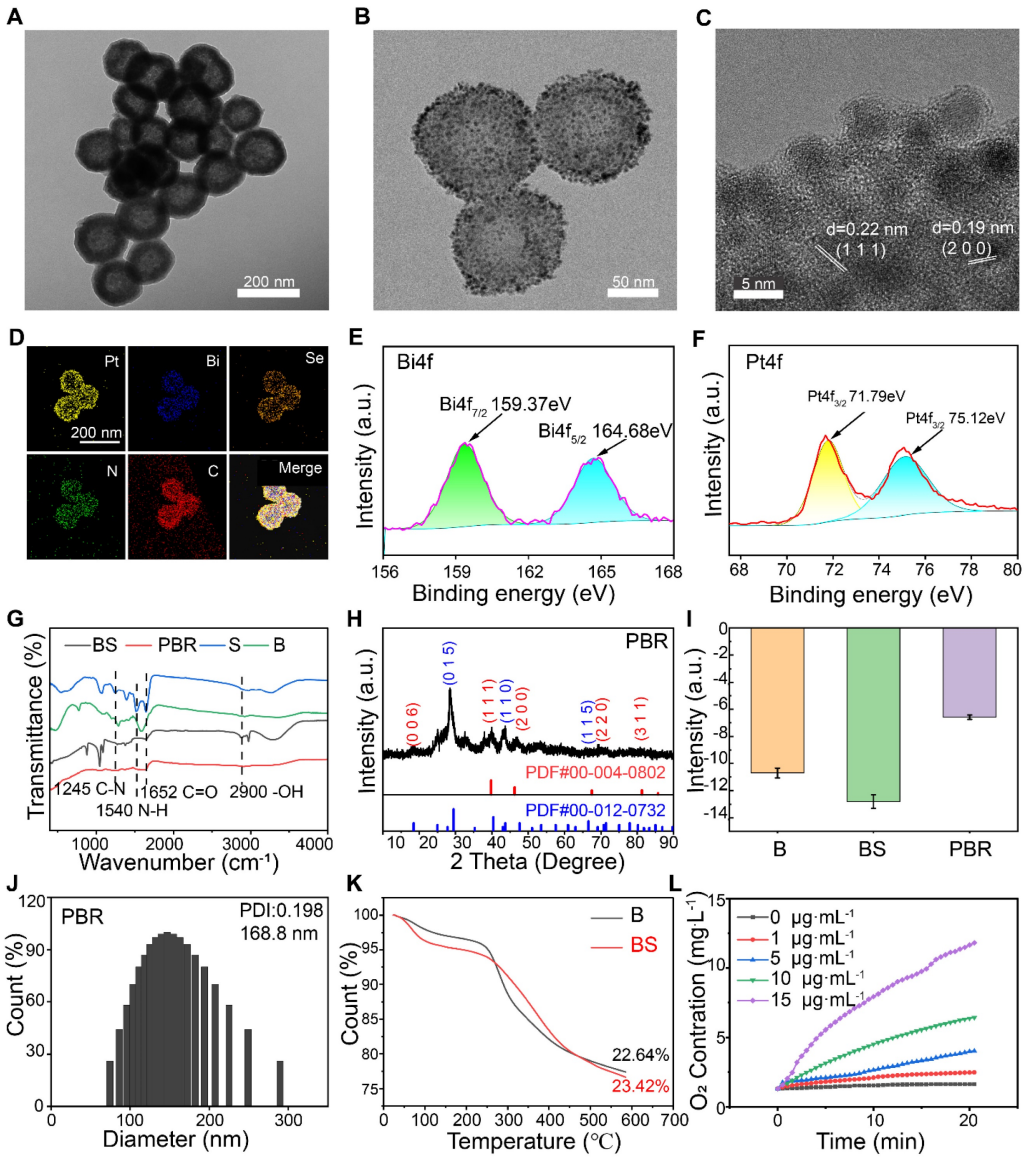
Characteristics of PBR. High-resolution TEM images of (A) Silk sericin containing Bi_2_Se_3_ (BS), (B) PBR, and (C) Pt on the surface of PBR; (D) Elemental mapping of PBR by TEM; X-ray photoelectron spectroscopy (XPS) spectra of (E) Bi 4f and (F) Pt 4f in PBR; (G) Fourier transform infrared spectra of S, B, BS, and PBR; (H) X-ray diffraction (XRD) pattern of PBR; (I) Zeta potential of B, BS, and PBR; (J) Hydrodynamic diameter of PBR measured using dynamic light scattering; (K) Thermogravimetric analysis results for B and BS; (L) Results of catalase-like (CAT) activity assay of PBR.

Bi_2_Se_3_ NPs containing silk sericin were initially synthesized using Bi_2_O_3_ as a self-sacrificing template, employing an improved solvothermal method 44. As shown in Figure 2A, Bi_2_Se_3_ containing sericin showed a more uniform morphology than bismuth selenide alone (Figure S1). Pt NPs were subsequently anchored to the surface of Bi_2_Se_3_ through the reduction method using sodium borohydride (Figure 2B). The cRGD-PEG-NH_2_ peptide-modified platinum-loaded Bi_2_Se_3_ NPs (Pt@Bi_2_Se_3_-RGD, PBR) were prepared using liquid phase synthesis 45. Transmission electron microscopy (TEM) revealed that Bi_2_Se_3_ loaded with Pt NPs exhibited a spherical shape, averaging at 160 nm, whereas Pt NPs averaged below 10 nm in size (Figure 2C). According to the HRTEM image, the spacings of the lattice were measured to be 0.22 and 0.19 nm, which were in agreement with the interplanar spacings of Pt (1 1 1) and Pt (2 0 0).

The coexistence of Pt, Bi, Se, C, and N in the hollow PBR nanoassembly was further confirmed through energy dispersive spectrometry mapping (Figure 2D), indicating the successful synthesis of Bi_2_Se_3_ NPs containing sericin. This result was also corroborated by X-ray photoelectron spectroscopy (Figure S2). The characteristic absorption bands at 159.37 and 164.68 eV in the Bi4f spectrum confirmed the existence of Bi^3+^(Figure 2E), whereas the absorption peaks at 71.79 and 75.12 eV in the Pt4f spectrum authenticated the presence of Pt^0^ (Figure 2F). In addition, the Se3d peak with a binding energy of 54.45 eV showed the presence of Se^2-^ (Figure S3). Fourier-transform infrared spectroscopy further demonstrated the characteristics of bismuth selenide NPs containing sericin (Figure 2G) with its characteristic amide peaks at 1652 cm^-1^ (amide I; due to C═O stretching), 1540 cm^-1^ (amide II; deriving from N-H in-plane bend), and 1245 cm^-1^ (amide III; deriving from C-N stretch), consistent with previous reports 42. Also, C═O stretching at 1652 cm^-1^ and -OH stretches detected at 2900 cm⁻^1^ in PBR confirmed the presence of sericin in PBR. Furthermore, X-ray diffraction (XRD) data of the PBR were consistent with those of Bi_2_Se_3_ (JCPDS No. 00-004-0802) and Pt (JCPDS No. 00-012-0732) (Figure 2H). Zeta potential of Bi_2_Se_3_ was -10.7 mV, which changed to -14.77 mV following the incorporation of silk sericin and to -6.58 mV after Pt loading and modification of the RGD peptide (Figure 2I). Dynamic light scattering demonstrated that the PBR particles had an average diameter of 168.8 nm (Figure 2J), and thermogravimetric analysis revealed a sericin content of 0.78% in Bi_2_Se_3_ (Figure 2K). We measured the concentration changes of dissolved O_2_ by incubating the PBR nanoassembly in 10 mM H_2_O_2_ solution to evaluate its catalase-like activity (Figure 2L). A notable elevation of dissolved O_2_ levels was observed with increased PBR concentration, indicating its potential to alleviate local hypoxia in tumors. Besides, terephthalic acid (TPA), which can produce fluorescent hydroxyl products when exposed to •OH, was used as a probe to detect hydroxyl radicals after irradiation. The fluorescence emission of TPA intensified under X-ray irradiation, and stronger fluorescence intensity was observed in the presence of PBR, verifying the production of •OH (Figure S4).

### Evaluation of the bioeliminable performance of PBR

**Figure 3:**
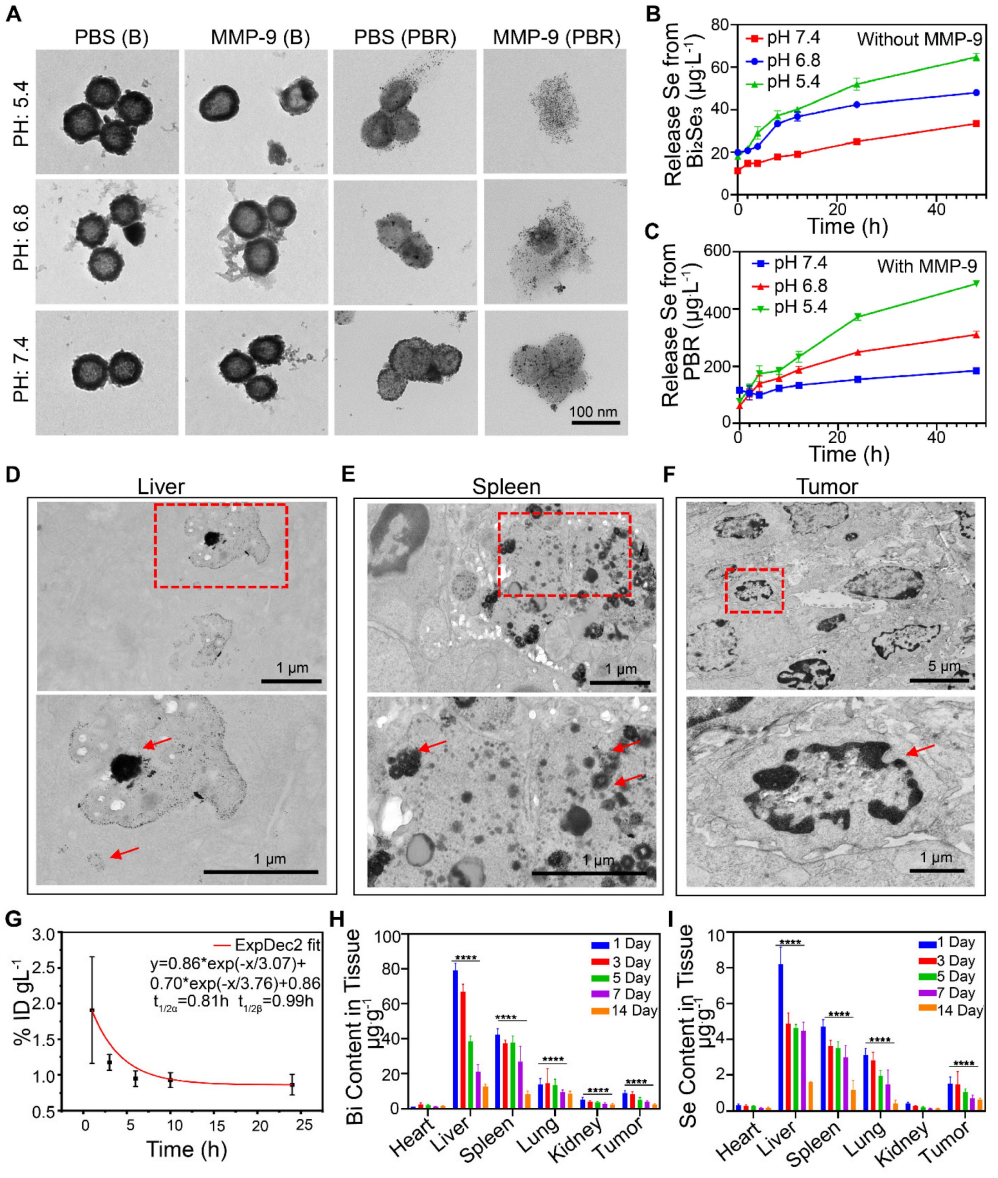
Biodegradation, pharmacokinetics, and biodistribution of PBR. (A) TEM images of PBR and B (Bi_2_Se_3_ without silk sericin) in PBS with or without MMP-9 at different pH values (5.4, 6.8, and 7.4); (B) Release of Se from Bi_2_Se_3_ at different pH levels without MMP-9 detection; (C) Release of Se from PBR at different pH values with MMP-9 detection; (D-F) TEM images of the liver (D), spleen (E), and tumor (F) Ultrathin sections 14 days after intravenous injection of PBR; (G) Pharmacokinetics of Bi content over 24 h after the intravenous injection of PBR; (H) Distribution of Bi in major organs 14 days after intravenous injection of PBR using ICP-MS; (I) Distribution of Se in major organs 14 days after intravenous injection of PBR compared to the 1-day group, *P < 0.05, **P < 0.01, ***P < 0.001 and ****P < 0.0001.

Morphological changes of the PBR were evaluated after 48 h of incubation under different pH conditions to investigate the biological elimination effect of the PBR nanoassembly. As displayed in Figure 3A, no significant morphological changes were observed in Bi_2_Se_3_ without silk sericin at pH 5.4, 6.8, and 7.4, representing the intracellular organelles, TME, and normal bodily fluid, respectively). Similarly, no alteration was observed after adding MMP-9. In contrast, Bi_2_Se_3_ containing silk sericin exhibited slight morphological changes in phosphate buffer (pH 7.4) and considerable morphological changes under acidic conditions. These changes manifested as collapsed structures and the detachment of Pt NPs from the Bi_2_Se_3_ surface. The presence of MMP-9 in a mildly acidic buffer (pH 6.8) separated most Pt NPs from the PBR, leading to a notable collapse of the spherical structure compared to the condition without MMP-9. Similarly, most Pt NPs were detached from PBR in the buffer devoid of MMP-9 when the pH was reduced to 5.4, resulting in a slight morphological collapse. Scattered Pt NPs were observed when MMP-9 was used with PBR in phosphate-buffered saline (PBS) (pH 5.4 or 6.8).

These results indicated that PBR could collapse into ionic and platinum NPs in an intracellular environment with acidic pH and MMP-9. The significant structural change observed implied that PBR has the potential to be eliminated by living organisms. To further validate its potential for biological elimination, we evaluated the biodegradation efficiency of PBR using cathepsin B, which is highly expressed in malignant tumors and accounts for 20% of lysosomal proteases 46. As shown in Figure S5, the platinum NPs detached from PBR after adding cathepsin B, resulting in a more pronounced spherical collapse. Moreover, the acidic pH reaction system was more conducive to structural collapse. Biodegradation products, including selenium, are essential trace elements for humans and animals and contribute to various biological functions related to antioxidant activity, anti-aging, immune enhancement, tumor prevention, and hormone metabolism 47-49.

Also, the biodegradation products Se and Bi in the supernatant were detected using inductively coupled plasma mass spectrometry (ICP-MS). Minimal Se (22.28 μg·L^-1^) and Bi (17.22 μg·L^-1^) were released from pure Bi_2_Se_3_ incubated in phosphate buffer at pH 7.4 over 48 h (Figures 3B and S6). At pH 5.4, the Se content change in 48 h was 46.75 μg·L^-1^, whereas that of Bi was 28.71 μg·L^-1^. This phenomenon was attributed to the dissolution of salt under acidic conditions. In contrast, PBR released 411.37 μg·L^-1^ Se when MMP-9 was added, which is almost 8.8 times higher than the pure Bi_2_Se_3_ at pH 5.4 (Figure 3C), and 353.5 μg·L^-1^ Bi was released from PBR at pH 5.4. These results demonstrated that PBR responds to pH and MMP-9 biodegradation by releasing selenium and bismuth ions.

The *in vivo* bioelimination of PBR was also investigated. After intravenous injection of 20 mg·kg^-1^ PBR into BALB/c mice bearing a 4T1 subcutaneous tumor for 14 days, ultrathin tissue slices of the liver, spleen, and tumor were observed by TEM (Figure 3D, 3E, and 3F). PBR exhibited high intracellular contrast in the liver, with small Pt NPs scattered near the PBR. Similarly, aggregated PBR was found in spleen vesicles. The morphology of PBR revealed broken nanoassemblies and detachment of Pt NPs from the PBR. Furthermore, no intact PBR was found in the tumor tissues. However, cancer cells suffered damage, such as nuclear condensation, indicating that the TME with low local pH and MMP-9 expression could accelerate the collapse of the PBR. These results suggested that PBR could collapse into ionic and platinum NPs in the intracellular environment with acidic pH and MMP-9. Although PBR exhibited bioeliminability in simulated *in vivo* microenvironments, its high dose and long-term effects in practical clinical applications require further investigation.

### Pharmacokinetics and biodistribution of PBR

We examined the *in vivo* distribution and biological elimination of the PBR. Initially, the circulation of PBR in the body after intravenous injection was determined by measuring the concentration of Bi in the blood at different time points using ICP-MS. The systemic circulation half-lives of the PBR nanoassembly during the distribution and elimination phases were 0.12 and 5.99 h, respectively (Figure 3G). The distribution of PBR was evaluated over two weeks following an intravenous injection of 20 mg·kg^-1^. The Bi and Se contents in the heart, liver, spleen, lungs, kidneys, and tumor tissues were quantified on days 1, 3, 5, 7, and 14 (Figure 3H, 3I). Bi and Se were detected by ICP-MS, with significant accumulation primarily observed in the reticuloendothelial system (liver and spleen). On day 1 post-injection, Bi was found in the liver (79.1 μg·g^-1^ of tissue), spleen (42.2 μg·g^-1^ of tissue), and lung (13.9 μg·g^-1^ of tissue), which had abundant resident macrophages for clearance of foreign matters from the body. Se concentrations were 8.22, 4.7, and 3.12 (μg·g^-1^ of tissue) in the liver, spleen, and lungs, respectively. Bi content in the tumor and kidney was 9.0 μg·g^-1^ and 5.3 μg·g^-1^, respectively, on day 1 after the injection. Se was also found in the tumor tissue (1.5 μg·g^-1^ of tissue) and kidney (0.4 μg·g^-1^ of tissue), demonstrating that PBR could be circulated to and reside in the tumor by the EPR effect and RGD-mediated active targeting before gradual clearance from the body via renal clearance. Bi and Se concentrations in the major organs decreased over time. By day 14, the Bi concentration had decreased to 12.59 in the liver, 8.53 in the spleen, and 8.63 in the lung μg·g^-1^, and the concentration of Se decreased to 1.6, 1.17, and 0.42 μg·g^-1^ in the liver, spleen, and lung, respectively. These observations suggested that PBR was gradually cleared from the body, which was consistent with the bioelimination observations of PBR *in vitro*.

### PBR-mediated radiosensitization *in vitro*

**Figure 4:**
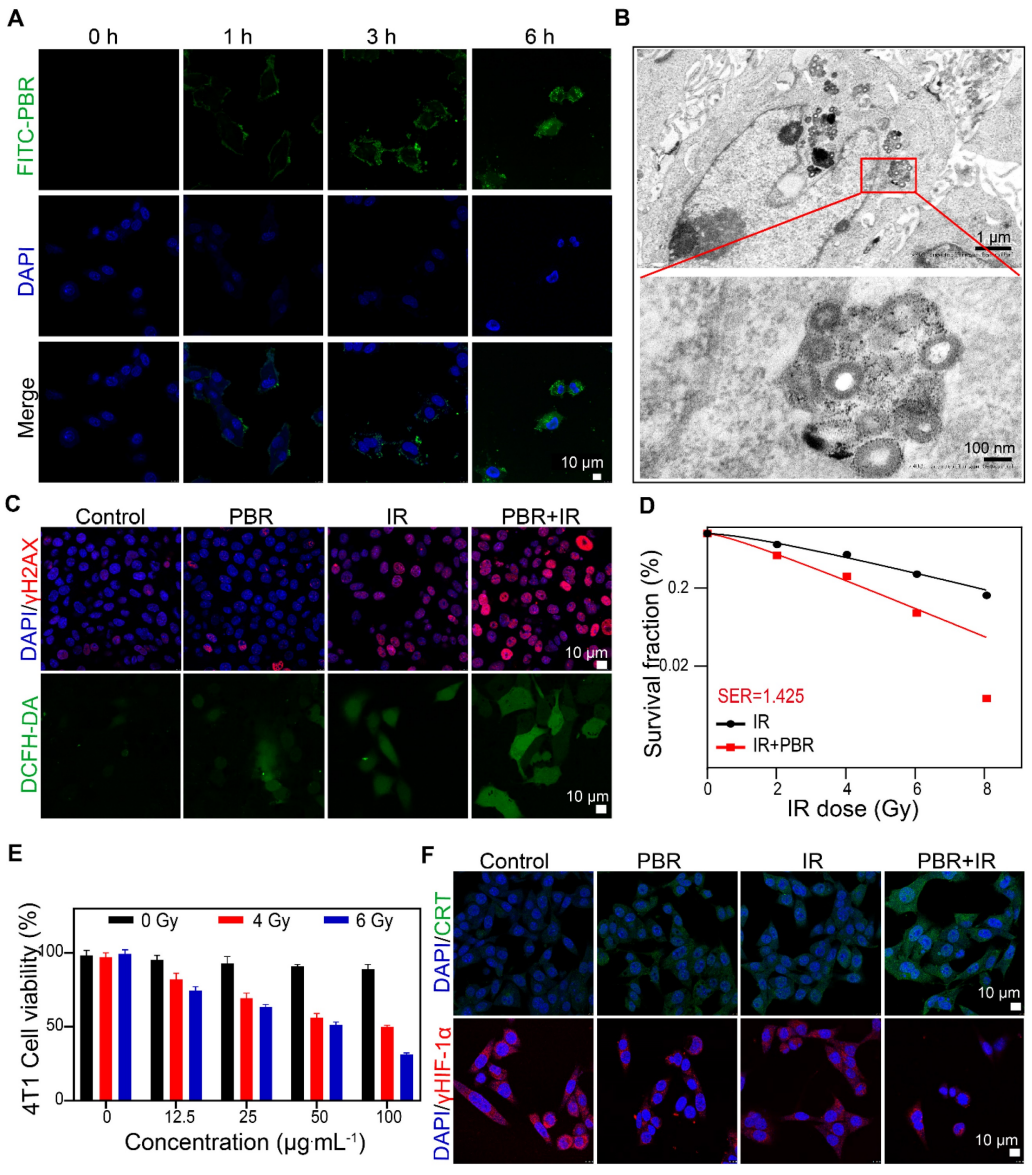
Synergy of PBR with radiotherapy *in vitro* (A) Intracellular uptake of FITC-labeled PBR by 4T1 tumor cells; (B) TEM images of ultrathin sections of 4T1 tumor cells after incubation with PBR at 37 °C for 6 h; (C) Evaluation of DNA damage using γH2AX, assessment of ROS using DCFH-DA; (D) Sensitization curve of 4T1 cells following treatment with different doses of radiation (PBR, 25 µg·mL^-1^); (E) Viability of 4T1 cells after different doses of radiation; (F) Immunofluorescence staining of CRT and HIF-1α markers. Statistical analysis was conducted using a *t*-test. Data are expressed as the mean ± SD. *P < 0.05, **P < 0.01, ***P < 0.001, and ****P < 0.0001.

We evaluated the endocytosis of PBR nanoassemblies in 4T1 tumor cells by laser scanning confocal microscopy. Fluorescein 5-isothiocyanate (FITC)-labeled PBR was internalized into the cytoplasm of cells, increasing fluorescence intensity over time (Figure 4A). TEM images of cells co-treated with PBR for 6 h confirmed that PBR was distributed near the nucleus (Figure 4B). The disintegration of the PBR structure was also observed, demonstrating its bioeliminability and indicating that intracellular enzymes and the acidic environment in lysosomes/endosomes gradually eliminated the PBR. Cellular internalization of PBR was visualized by staining with LysoTracker Red (Figure S7). Initially, increased FITC fluorescence in cells showed gradual overlap with the LysoTracker signals over the incubation time of up to 3 h, suggesting the endocytosis of nanoparticles transported by lysosomes. No fluorescence overlaps were observed after 6 h, implying that the PBR was released and diffused from lysosomes to the cytoplasm. PBR-treated 4T1 cells were subjected to various endocytosis inhibitors to understand the mechanism. As displayed in Figure S8, among other endocytosis inhibitors, including nystatin (caveolar-mediated), amiloride (macropinocytosis-mediated), and CPZ (clathrin-mediated), Me-β-CD had the highest efficiency in inhibiting PBR endocytosis, implying that the PBR was internalized via the lipid-raft-mediated endocytosis pathway.

Next, we investigated the efficacy of radiotherapy sensitization of PBR. The DNA damage induced by radiotherapy in 4T1 cells was evaluated by γ-H2AX immunofluorescence staining. As shown in Figure 4C and S9, a notable elevation was observed in the fluorescence intensity of red γ-H2AX foci in cells treated with PBR and irradiation (IR) (irradiation with Co^60^, γ-ray 4 Gy) compared to those exposed to radiation alone. DCFH-DA was used to detect intracellular ROS levels. The highest intensity of green fluorescence was observed in the IR+PBR group compared with the control group, indicating that PBR achieved radiosensitization by increasing ROS levels in tumors. Colony formation assays were conducted to validate the radiosensitization effect of PBR *in vitro*. When the survival rate of cancer cells treated with IR or IR + PBR was calculated based on the number of colonies at different radiation doses, a sensitivity enhancement ratio (SER) of 1. 425 was observed (Figure 4D, S10). Live-dead staining of 4T1 after different treatments also provided evidence for the radiosensitizing function of PBR (Figure S11).

Furthermore, the CCK-8 assay was performed to evaluate the effect of varying concentrations of PBR on 4T1 (Figure 4E). PBR did not exert a notable inhibitory effect on 4T1 cells at concentrations of up to 100 μg·mL^-1^. In contrast, the combination of PBR and IR resulted in higher growth inhibition of 4T1 cells. The extent of inhibition depended on the PBR concentration and radiation dose. The cell survival rate decreased to 50% at an IR dose of 4 Gy and concentration of 100 μg·mL^-1^; it further decreased to 31% when the IR dose was 6 Gy. To minimize the toxicity of irradiation, we selected 4Gy as the irradiation dose for subsequent cell experiments. These findings suggested that PBR nanoassemblies could accumulate within 4T1 cells and enhance radiation effects by increasing DNA damage and inhibiting cell proliferation.

HIF-1α immunofluorescence staining was used to evaluate the intracellular hypoxia states. The fluorescence intensity of HIF-1α in PBR-treated 4T1 cells was relatively weaker than that of untreated cells. This was attributed to the ability of PBR to catalyze H_2_O_2_ in the TME, consequently alleviating tumor hypoxia (Figure 4F, S12). Furthermore, Western blotting analysis demonstrated reduced HIF-1α expression in PBR-treated cells (Figure S13). This was consistent with the cell immunofluorescence staining results.

It has been reported that nanomedicine-enhanced cancer treatment could induce immunogenic cell death (ICD), possibly activating adaptive immune responses by releasing danger-associated molecular patterns into the TME 50. Under PBR-mediated sensitization, the expression of the ICD marker calreticulin (CRT) was significantly increased on the cell membrane (Figure 4F), and another marker, high-mobility group box 1 (HMGB1), was released from the nucleus (Figure S14). Moreover, the lowest intracellular adenosine triphosphate (ATP) levels were observed as determined by an ATP assay kit. (Figure S15). These findings suggested that PBR-mediated radiotherapy effectively induces ICD in 4T1 cells.

### PBR induces apoptosis via the Areg/Egfr/Bcl-2 pathway

**Figure 5:**
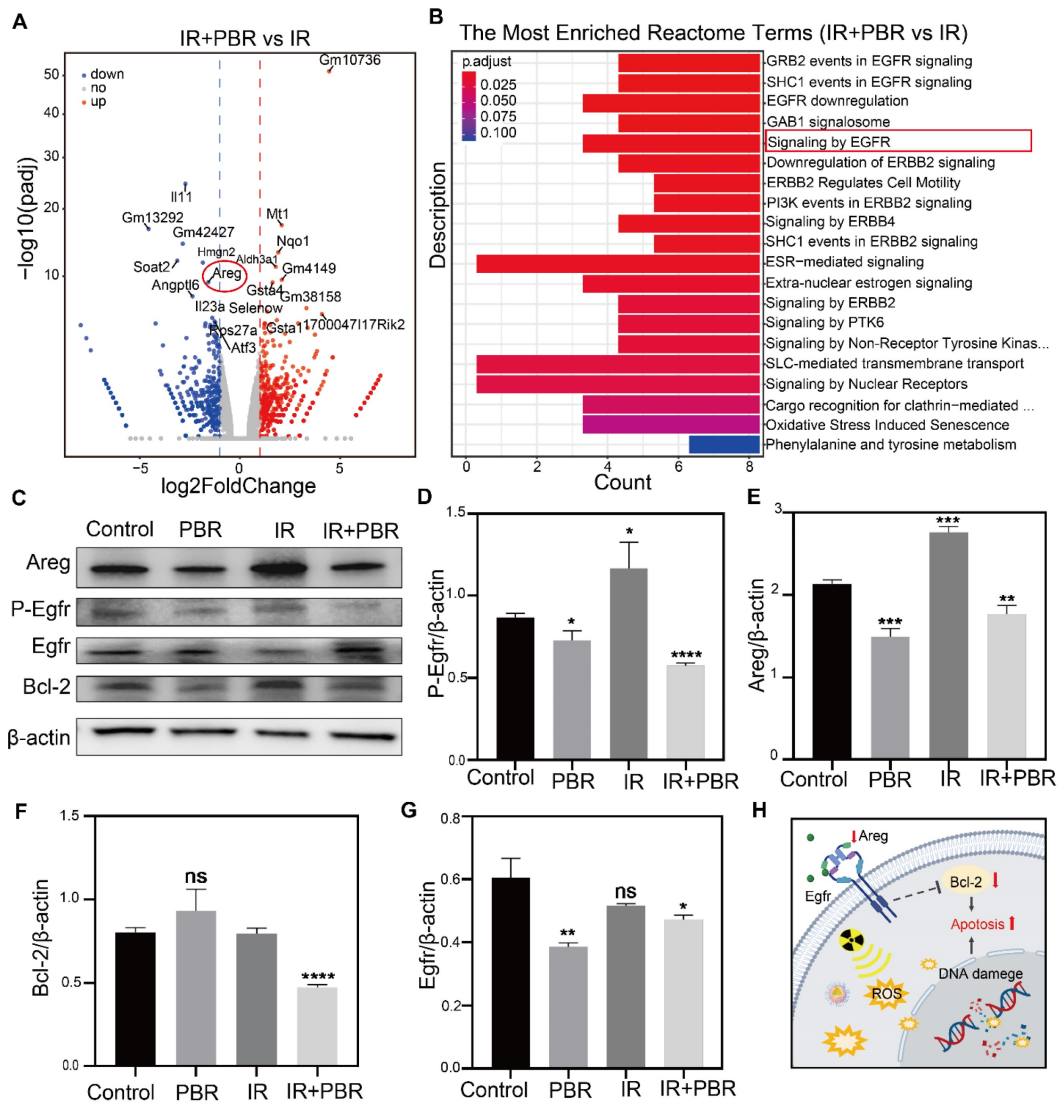
Mechanisms underlying PBR-mediated radiosensitization. (A) Volcano plot showing significant differences in the expression of genes between the PBR+IR treatment and IR groups; (B) Reactome enrichment analysis showing gene function; (C) Changes in the expression of Areg, Egfr, p-Egfr and Bcl-2 in 4T1 cells following different treatments determined by Western blotting; (D-G) Semi-quantitative analysis demonstrating expression levels of Areg, Egfr, p-Egfr and Bcl-2 proteins; (H) Illustration of the effect of PBR on the Areg/Egfr/Bcl-2 signaling pathway; Statistical analysis was performed using a *t*-test. Data are presented as the mean ± SD. *P < 0.05, **P < 0.01, ***P < 0.001 and ****P < 0.0001.

Since *in vitro* evidence indicated PBR's potential for radiosensitization, we conducted transcriptome sequencing to investigate the underlying mechanisms. Comparative analysis of gene expression was performed between the IR+PBR and IR groups. In total, 1,135 differentially expressed genes were identified in different groups. Of these, the expression of 618 and 517 genes was upregulated and downregulated, respectively (Table S1). The volcano plot illustrated notable disparities in gene expression between the two groups (Figure 5A). Reactome pathway enrichment analysis indicated that the epidermal growth factor receptor (Egfr) pathway involved in regulating cellular processes, such as the cell cycle, proliferation, differentiation, and survival 51,52, was most significantly downregulated (Figure 5B). Egfr is a tyrosine kinase receptor involved in fundamental signaling pathways and is, therefore, a major target in oncology 53, 54. The volcano plot also illustrated significant downregulation of Areg, an oncogenic factor that competes with Egf for binding to Egfr 55. (Figure 5a, Table S2). Previously, the Areg/Egfr/Bcl-2 pathway has been shown to impede the proliferation and migration of tumor cells 56,57. The real-time quantitative PCR (qPCR) analysis revealed that PBR treatment downregulated Areg expression (Figure S16). Western blot analysis also showed significant downregulation of Areg when treated with PBR+IR compared with the IR alone group (Figure 5C-5G). PBR affects apoptosis through the Areg/Egfr/Bcl-2 pathway. Consequently, the binding with the Egf receptor was reduced, resulting in the downregulation of phosphorylated Egfr (p-Egfr) expression. Areg also regulates the anti-apoptotic protein Bcl-2 via Egfr 58,59. We observed Bcl-2 and Caspase3 downregulation following treatment with PBR+IR compared with the IR group (Figures 5C, S17). These results provided evidence that PBR enhances apoptosis in tumor cells by affecting the Areg/Egfr/Bcl-2 signaling pathway (Figures 5H).

### *In vivo* imaging

**Figure 6:**
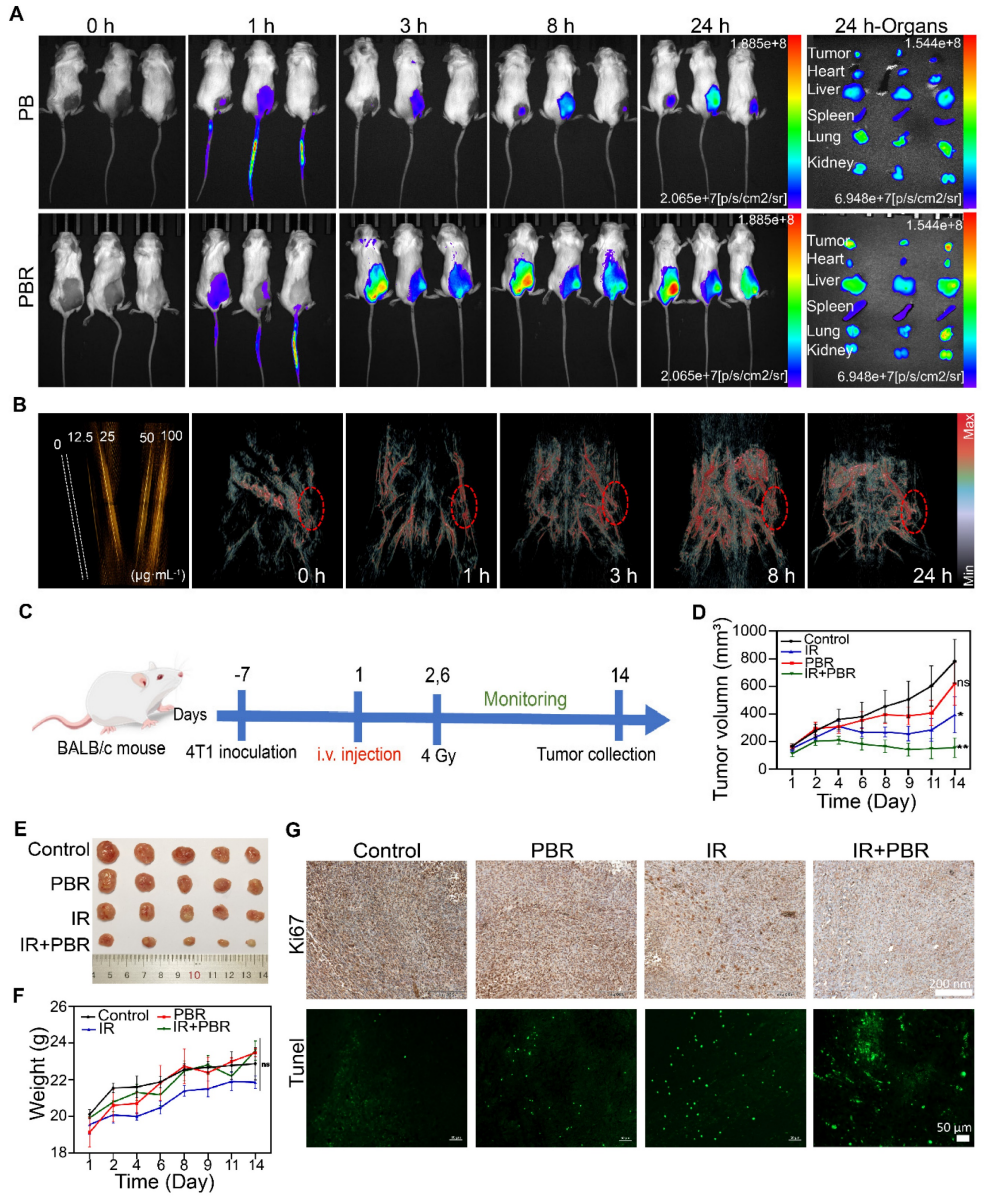
*In vivo* imaging and treatment of 4T1 tumor-bearing mice following PBR-mediated radiosensitization. (A) Fluorescence imaging of 4T1 tumor-bearing mice injected with IR783-labeled PBR or PB (20 mg·k^-1^) and fluorescence distribution in excised organs and tumors 24 h after injection; (B) PA signals of different concentrations (0, 12.5, 25, 50, and 100 μg·mL^-1^) of PBR in aqueous solution and photoacoustic imaging of tumor-bearing nude mice after injection with PBR (20 mg·k^-1^) under 808 nm laser excitation; (C) Schematic diagram of the treatment process; (D) Tumor volume growth curves under different treatment methods; (E) Tumor photographs after 14 days of treatment; (F) Statistical analysis of body weight of different groups; (G) Histological analysis of tumor sections by Ki67 immunohistochemical and Tunel immunofluorescence staining on day 14. Statistical analysis was performed using a* t*-test. Data are presented as the mean ± SD. *P < 0.05, **P < 0.01.

The precise delivery of nanoparticles is a critical strategy for enhancing therapeutic efficacy. Therefore, we used fluorescence and photoacoustic (PA) imaging to investigate PBR's tumor-targeting capability and biodistribution in a subcutaneous 4T1 tumor model. Stronger fluorescence accumulation at the tumor site could be observed in mice after intravenous injection of IR783-labeled PBR than those injected with IR783-labeled PB. Semi-quantitative analysis showed 26.4% fluorescence intensity for PBR and 15.3% for PB at the tumor site, indicating that modifying the RGD peptide could improve the efficiency of tumor site targeting (Figure 6A). The main organs and tumors were collected for fluorescence imaging 24 h after injection (Figure S18); the signal intensity in the tumor tissue was stronger than in other tissues. PBR also generated considerable photoacoustic signals under 808 nm laser stimulation compared to water (Figure 6B, S19), indicating that it functions as a PA imaging contrast agent due to its near-infrared absorption properties. Therefore, we intravenously injected PBR and detected the photoacoustic signals in 4T1 tumor-bearing nude mice at different time points. At 8 h post-injection, strong photoacoustic signals were visualized in blood vessels of the tumor region under 808 nm laser excitation (Figure S19), and the signal did not decrease significantly at 24 hours, confirming its tumor-targeting ability. These results demonstrated that we successfully engineered PBR that can accurately target tumor sites, enhancing radiotherapy efficacy.

### PBR mediated radiosensitization *in vivo*

Given PBR's significant radiosensitization effects and tumor-targeting ability *in vitro*, we evaluated its performance in a subcutaneous 4T1 tumor-bearing mouse model. Tumor-bearing mice were randomly assigned to four groups (five per group): control, IR, PBR, and IR+PBR. PBR was injected on day 0 (with PBS injection as a control), and the tumor region received two irradiation doses (Figure 6C). To minimize radiation exposure while effectively inhibiting tumor growth, we set the total irradiation dose to 8 Gy, delivering two fractions of 4 Gy each on days 1 and 6. PBR treatment showed a modest tumor inhibition rate of 20.70% (Figures 6D, 6E). The IR group exhibited a tumor inhibition rate of 49.42%, whereas the PBR + IR group achieved the highest tumor inhibition rate of 75.16% at the end of the treatment. Consistent with the tumor growth data, tumor weight analysis confirmed the antitumor effects of PBR (Figure S20). The weight gain in the IR group was slower than in other groups (Figure 6F), which might be attributed to the bioeliminable PBR. In a previous study, selenium has been shown to regulate oxidative stress 60. Therefore, we evaluated superoxide dismutase (SOD) activity in different treatment groups (Figure S21). Serum SOD activity in the PBR-treated group was higher than that in the IR-treated group. This observation was consistent with a report that selenium-containing nanomaterials degrade *in vivo* to selenium in the bloodstream, enhancing the immune response and increasing SOD activity, protecting normal tissues from radiation damage 61. We investigated the therapeutic effects of the different treatments using H&E, Ki67, and Tunel staining, focusing on tumor cell proliferation and apoptosis. As shown in Figure 6G, the IR+PBR group exhibited significantly more tumor cell necrosis than the control group by H&E staining (Figure S23). Ki67 staining revealed a marked reduction in Ki67-positive signals in the IR+PBR group, whereas Tunel staining showed enhanced green fluorescent signals for markers of cell damage in this group. These observations indicated that combined treatment effectively inhibited tumor cell proliferation and induced apoptosis (Figure S22).

### PBR-mediated radiotherapy sensitization synergistic with PD-L1 blockade

**Figure 7:**
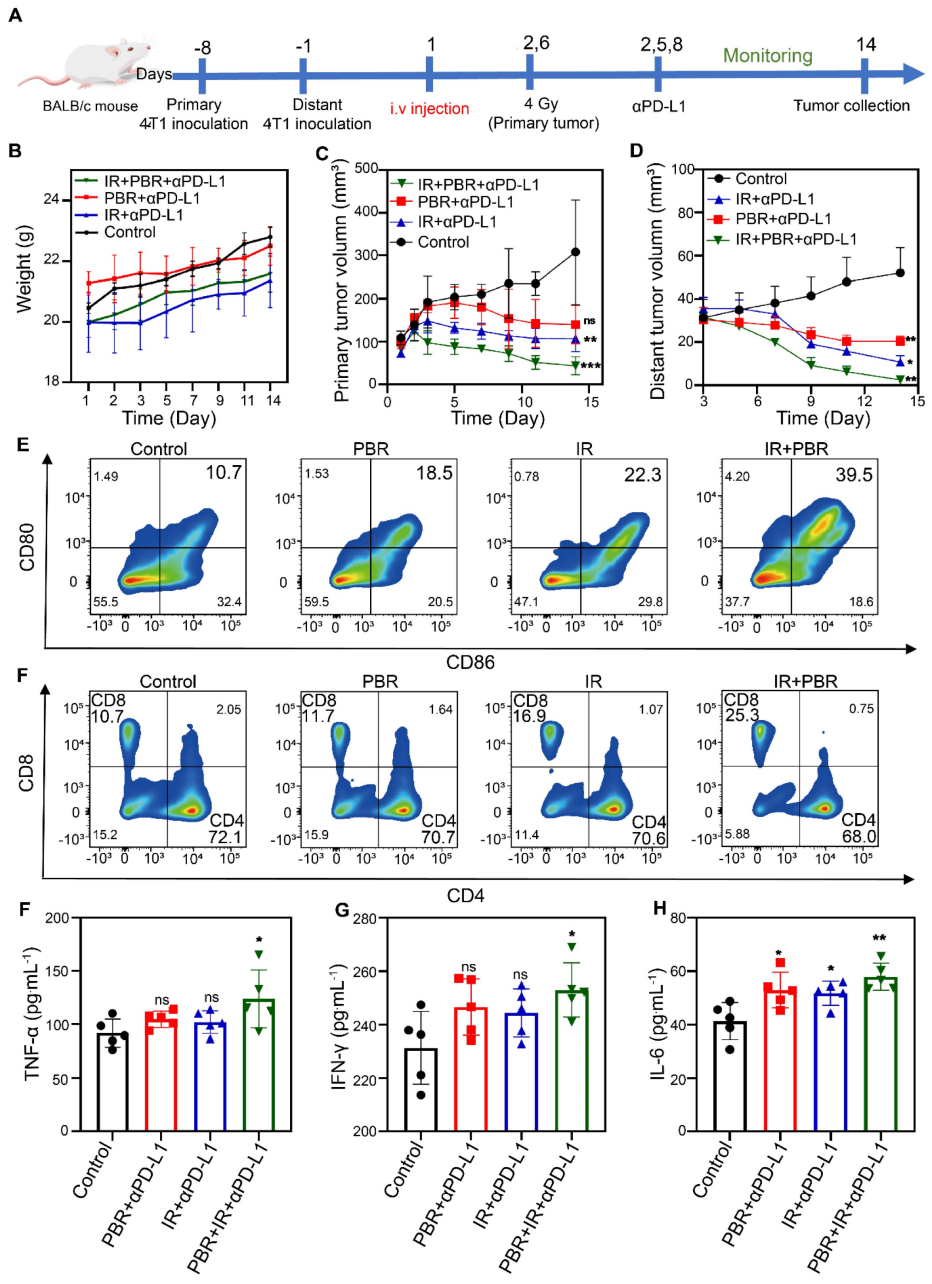
PBR-mediated radiopotentiation combined with PD-L1 antibody therapy for bilateral tumors. (A) Schematic representation of the experimental procedure for bilateral tumors; (B) Body weight curves of female 4T1 tumor-bearing BALB/c mice during treatment; (C) Growth curves of primary tumors during various treatment; (D) Growth curves of distant tumors; (E) Flow cytometric analysis of the maturity characteristics of dendritic cells (CD80^+^ and CD86^+^) in the lymph nodes; (F) T cells in the spleen from each treatment group (CTL: CD3^+^, CD4^+^, and CD8^-^; Th cells: CD3^+^, CD4^-^, and CD8^+^); (G-I) Changes in serum levels of cytokines (G) TNF-α, (H) INF-γ, and (I) IL-6 from different treatment groups. Statistical analysis was performed using a *t*-test. Data are presented as the mean ± SD. *P < 0.05 and **P < 0.01.

Our preliminary findings indicated that PBR exhibits significant radiosensitization effects and mediates ICD activation. We established a bilateral subcutaneous tumor model to investigate the inhibitory effects of PBR-mediated radiosensitization combined with PD-L1 blockade on distal tumors. To this end, 4T1 cells were inoculated into the right flank of BALB/c mice on day 8 to form primary tumors and then inoculated into the left flank of the mice on day 1 to form distal tumors. Mice were randomly grouped, and treatments were administered according to the experimental plan (Figure 7A). The mice received intravenous injections of PBR on day 1. Anti-PD-L1 (75 μg·kg^-1^) was administered via intraperitoneal injection on days 2, 5, and 8, whereas the primary tumors received a dose of 4 Gy radiation on days 2 and 6. No significant changes in body weight were observed after two treatment cycles, which was consistent with the treatment phase of the 4T1 subcutaneous tumor model (Figure 7B). In addition, the PBR + aPD-L1 and IR + aPD-L1 groups showed slower tumor growth than the control group. The tumor inhibition rates in the PBR + aPD-L1 group were 50.28% for primary tumors and 81.66% for distal tumors. Similarly, the IR + aPD-L1 group had inhibition rates of 64.06% and 79.97% for primary and distal tumors. The IR + PBR + aPD-L1 group displayed the most effective tumor growth inhibition, with 85.13% and 95.05% rates for primary and distal tumors (Figures 7C, 7D, S24, S25).

We assessed the percentage of mature dendritic cells (DCs), identified as CD80^+^ and CD86^+^, in the lymph nodes near the primary tumor (Figure 7E, S26) on day 5 and verified the PBR synergy with radiotherapy-induced immune responses in mice. The frequency of mature DCs in the PBR group increased by approximately 7.8%, whereas that in the IR group increased by about 11.6%. In contrast, the IR+PBR group exhibited a 28.8% increase in mature DCs, indicating that the antigens released by PBR with IR through ICD could effectively stimulate DC maturation. We also evaluated the number of activated cytotoxic T lymphocytes in mouse spleens to elucidate further the mechanism of antitumor immunotherapy mediated by PBR synergized with IR. The PBR and IR treatment groups recruited 1% and 6.2% more tumor-infiltrating CD8^+^ T lymphocytes (CD3^+^ and CD8^+^) than the untreated group. Moreover, the IR+PBR treatment group recruited 14.6% more CD8^+^ T lymphocytes than the control group (Figure 7F, S26). Additionally, we measured the serum levels of immune-related cytokines after different treatments using enzyme-linked immunosorbent assays of tumor necrosis factor-alpha (TNF-α), interleukin-6 (IL-6), and interferon-gamma (IFN-γ). Levels of these pro-inflammatory factors were elevated following IR + PBR + aPD-L1 treatment (Figures 7G, 7H, and 7I). These results indicated that PBR-mediated radiosensitization could synergize with the PD-L1 immune blockade to suppress distal tumors.

### Biocompatibility of PBR

The *in vivo* toxicity of PBR was comprehensively and systematically evaluated. Initially, PBR was co-cultured with normal cells (RLE-6TN cells) for 48 h. Varying PBR concentrations (200, 100, 50, 25, 12.5, and 0 μg·mL^-1^) did not induce significant cell death or exhibit cytotoxicity (Figure S27. Furthermore, the 200 μg·mL-1 PBR hemolysis rate was below 5%, indicating no hemolysis induction by PBR in tumor-bearing mice (Figure S28). Furthermore, the acute toxicity of PBR was evaluated in BALB/c mice. No abnormal behaviors, such as fur loss or mortality, were observed in the animals over the 14-day observation period following the intravenous injection of varying doses of PBR (Figure S29). No differences in body weight were observed between the treated and untreated groups. In addition, no significant discrepancies were identified in the complete blood count tests (Figure S30). Histological analysis revealed the absence of discernible indications of cellular necrosis or tissue damage in the heart, liver, spleen, lungs, and kidneys across all experimental groups (Figure S31). These results indicated a favorable biosafety PBR profile in tumor therapies.

## Conclusion

In this study, we synthesized a bioeliminable PBR nanoassembly as a radiosensitizer guided by photoacoustic imaging. An improved solvothermal method and a series of modifications were employed. Our approach aimed to achieve radiosensitization and amplification of antitumor immune responses by a bioeliminable PBR nanoassembly. PBR exhibited catalytic activity reminiscent of catalase, effectively decomposing hydrogen peroxide to generate oxygen, alleviating the significant hypoxic conditions typically associated with the TME, which is beneficial for radiotherapy. Furthermore, PBR downregulated Areg expression, inhibiting the Egfr signaling pathway and inducing apoptosis in tumor cells. PBR-mediated radiosensitization also effectively led to immunogenic cell death in cancer cells, operating synergistically with PD-L1 therapy to promote the suppression of primary and distal tumors. Additionally, the bioeliminablility of PBR alleviated the toxicity associated with its accumulation in tissues and mitigated the adverse effects of radiotherapy. This novel PBR sensitizer demonstrated significant potential for tumor-targeted therapy and synergistic treatment approaches.

## Materials and Methods

### Synthesis of PBR

Bi(NO_3_)_3_·5H_2_O, NaOH, and PVP were added to 25 mL of EG at a molar ratio of 61:135:531. The mixture was thoroughly stirred before it was heated to 150 ℃ and allowed to react for 3 h. Subsequently, the product was transferred to a centrifuge tube, and an appropriate amount of deionized water was added. After the sample was centrifuged for 8 min at 14,000 rpm, the supernatant was discarded. The precipitate was centrifuged and washed three times to obtain bismuth oxide (Bi_2_O_3_). AA, Na_2_SeO_3_, and Bi_2_O_3_ were dissolved in 10 mL of deionized water at a molar ratio of 170:21:160. SS (0.25 g) was then added. The mixture was thoroughly stirred, heated to 150 ℃, and allowed to react for 12 h. The product was centrifuged and washed three times to obtain SS-containing bismuth selenide (Bi_2_Se_3_-SS, BS). Bismuth selenide (Bi_2_Se_3_, B) was synthesized by using the above procedure. PVP, H_2_PtCl_3_, and the previously obtained bismuth selenide were dissolved in 50 mL of deionized water at a molar ratio of 45:15:763. Next, 30 mL of NaBH_4_ solution (4.65×10^-4^ mol·mL^-1^) was slowly dropped into the mixture while stirring in the dark for 6 h. The product was centrifuged and washed three times to obtain platinum-loaded bismuth selenide (Pt@Bi_2_Se_3_, PB). EDC and NHS were dissolved in 10 mL of deionized water at a molar ratio of 193:174. Next, 0.5 mg of the prepared Pt@Bi_2_Se_3_ was added. The mixture was stirred in the dark at 25 ℃ for 0.5 h. Next, cRGD-PEG-NH_2_ (0.5 mol·mL^-1^) was added, and the mixture was stirred in the dark at room temperature for 10 h. The product was then dialyzed in deionized water to obtain a cRGD-PEG-NH_2_-modified bismuth selenide nanoassembly (Pt@Bi_2_Se_3_-RGD, PBR).

### Enzymatic capacity

The CAT-like activity of PBR was assessed by recording concentration changes of the dissolved oxygen in different PBR concentrations (0, 1, 5, 10, and 25 µg·mL^-1^) with 5 mM H_2_O_2_ using a dissolved oxygen meter (JPB-607A, Shanghai INESA Scientific Instrument, China). Hydroxyl radicals can oxidize terephthalic acid (TPA) to produce fluorescent products; thus, TPA is often used to detect the presence of hydroxyl radicals. We used TPA to evaluate the •OH radicals produced by PBR with irradiation. Specifically, 0.5 mM TPA reagent was mixed with PBR (25 μg·mL^-1^), providing irradiance when needed (4 Gy). The fluorescence spectrum of each product was recorded using a fluorescence spectrophotometer after continuing the reaction for 30 min.

### *In vitro* modeling of bioeliminable properties of PBR

The *in vitro* bioeliminable properties of PBR were evaluated. Specifically, 1 mg·mL^-1^ PBR was added to PBS at different pH values. Matrix metalloproteinase-9 (MMP-9, 0.1 µg·mL^-1^) was added to simulate the *in vivo* enzyme-containing microenvironment and the solutions were placed on a shaking incubator at 37 °C, 1500 rpm for 48 h. Finally, the morphologies of the treated PBR and B NPs were observed using TEM. We also added 0.1 µg·mL^-1^ Cathepsin B to different pH values of PBS to treat PBR. Se and Bi elements released from PBR and B in different pH PBS were detected using ICP-MS. If necessary, MMP-9 was added.

### *In vivo* biodistribution of PBR

4T1 tumor-bearing mice were intravenously injected with 20 mg·kg^-^¹ PBR. At various time points, three mice were randomly selected for euthanasia. The major organs and tumor tissues were then weighed and dried. The tissues were completely dissolved in aqua regia, and the concentrations of Bi and Se were determined using ICP-MS. Next, Bi and Se contents in different tissues were calculated. Biological electron microscope observations of liver, spleen, and tumor tissues were made on Day 14 post-injection.

### Pharmacokinetics of PBR

4T1 tumor-bearing mice were intravenously injected with 20 mg·kg^-^¹ PBR. Ten microliters of tail blood were collected from the mice at various time points post-injection to assess the circulation time of PBR *in vivo*. Following thorough dissolution in 1 mL of nitric acid and digestion using a microwave digestion system, the samples were diluted with deionized water, and the concentrations of Bi and Se were measured using ICP-MS.

### Biocompatibility of PBR

The biocompatibility of PBR was evaluated using rat type II alveolar epithelial cells (RLE-6TN) as representatives of normal tissue cells. RLE-6TN cells were cultured in 96-well plates, different concentrations of PBR were added, and the cells were incubated for 48 h. Cell viability was then assessed using the CCK-8 assay. Whole blood from two healthy mice was placed in anticoagulant tubes, centrifuged at 2000 rpm for 5 min, and washed thrice with PBS to obtain a red blood cell suspension (4 % in PBS). Next, different concentrations of PBR were mixed with the red blood cell suspension and allowed to stand at 37 °C for 3 h. Hemolysis was measured using a microplate reader to assess the extent of red blood cell lysis in the supernatant. Different dosages of PBR (0, 12.5, 25, 50, and 100 mg·kg^-^¹) were intravenously injected into female BALB/c mice to evaluate its toxicity. The general health of mice was monitored, and their body weights were recorded daily. After 14 days, blood was collected from the mice, and various parameters were analyzed using an automatic hematology analyzer (URIT-5160 Vet; URIT, China). The major organs of these mice were embedded in paraffin, sectioned, and subjected to hematoxylin-eosin (HE) staining. Pathological changes were observed using a fluorescence microscope (Leica, Wetzlar, Germany).

### Cell endocytosis of PBR

4T1 cells were treated with FITC-labeled PBR (25 µg·mL^-^¹) in confocal plates and incubated at 37 °C in a 5 % CO₂ incubator for 0, 1, 3, and 6 h. Next, the cells were thoroughly washed with PBS to remove free PBR, fixed with paraformaldehyde, and treated with an anti-fade DAPI reagent. Cells were observed and imaged using a laser-scanning confocal microscope. To further verify whether PBR could be internalized by the cells, 4T1 cells were treated in T25 culture flasks with PBR (25 µg·mL^-^¹) for 6 h. After treatment, cells were thoroughly washed with PBS to remove free PBR and exposed to 4 Gy of irradiation if necessary. The cells were rewashed with PBS, digested with trypsin, and fixed with 1.5 mL of 2 % glutaraldehyde solution. Ultrathin sections were obtained using an ultramicrotome, stained, and imaged using a transmission electron microscope (HT7800; Hitachi, Japan). To investigate the lysosomal co-localization and escape, 4T1 cells were seeded in a 35-mm Petri dish at a density of 3×10^4^ cells. Upon reaching a culture confluency of 50%-70%, cells were further incubated with PBR (25 μg·mL^-1^) for various time periods. Then, the cells were stained with Hoechst33342 (1 μg·mL^-1^) for 5 min and Lyso-Tracker Red (50 nM) for 45 min. Finally, fluorescence images of stained cells were captured using a laser-scanning confocal microscope. To explore the underlying mechanism of cellular uptake of PBR, 4T1 cells were pretreated with Nystatin (50 μg·mL^-1^), Amiloride (2 mM), CPZ (20 μg·mL^-1^), and Me-β-CD (0.5 mM), followed by incubation with PBR (25 μg·mL^-1^) for 6 h. The cellular uptake efficiency under various conditions was analyzed using flow cytometry.

### PBR-mediated radiosensitization* in vitro*

We assessed the DNA damage induced by RT on 4T1 cells using γ-H2AX immunofluorescence staining. Briefly, 4T1 cells were cultured overnight in a confocal dish. They were then treated with PBR (25 µg·mL^-^¹) for 6 h. After treatment, the cells were thoroughly washed with PBS to remove free PBR and exposed to 4 Gy of irradiation if necessary. Then, the cells were further incubated for 24 h. Next, cells were fixed with paraformaldehyde and washed with PBS. Subsequently, the cells were permeabilized using Triton-X100 and treated with the blocking buffer. The cells were then incubated with γ-H2AX antibody and labeled with the appropriate fluorescent secondary antibodies. Finally, images of the cells were acquired through confocal microscopy. DCFH-DA was used to assess intracellular ROS levels in different treatment groups. 4T1 cells were seeded in 6-well plates and allocated to four groups: control, irradiation (IR), PBR, and IR + PBR. After co-incubation with PBR (25 µg·mL^-^¹) for 6 h, the cells were processed with DCFH-DA according to the manufacturer's instructions. 4T1 cells were seeded in 6-well plates and allocated to the same four groups. After co-incubation with PBR (25 µg·mL^-^¹) for 6 h, the cells were processed with Calcein/PI Cell Activity and Cytotoxicity Assay Kit. Fluorescence analysis was carried out using confocal microscopy. We performed colony formation experiments to validate the radiotherapy sensitization efficiency of PBR *in vitro*. 4T1 cells were seeded at a density of 500 cells per well in 6-well plates. Following 6 h coculture with 25 µg·mL^-^¹ PBR, the cells were washed, and fresh medium was added. The cells were then irradiated at doses of 0, 2, 4, 6, and 8 Gy and cultured for an additional 10 days. After that, the cells were fixed with glutaraldehyde, stained with crystal violet solution, and allowed to air dry at room temperature. Stained cell colonies were photographed and counted using a digital camera. The sensitive enhancement ratio (SER) of PBR was determined by curve fitting of the surviving fraction. CCK-8 assays were performed to evaluate the toxicity of PBR on 4T1 and A549 cells. Cells were cultured in 96-well plates following standard procedures, cocultured with various concentrations of PBR for 6 h, exposed to irradiation, and subsequently cultured for 24 h. Next, we employed immunofluorescence staining and Western blotting to evaluate the changes in the expression of relevant molecules. After following the previous processing steps, cells were treated with anti-HIF-1α, anti-HMGB1, anti-CRT, confocal microscopy, or Western blotting, and the results were recorded. Intracellular ATP levels were measured using an ATP assay kit (Beyotime).

### Apoptosis induction by PBR via the Areg/ Egfr/ Bcl-2 pathway

First, we sequenced and analyzed the transcriptomes of IR+PBR and IR-treated 4T1 cells. The cells were grown in 6-well plates, exposed to PBR for 6 h, and irradiated at a dose of 4 Gy. Cells were collected after 24 h incubation. Total RNA was extracted using TRIzol reagent (TaKaRa Biotechnology) for Q-PCR analysis. Quantitative PCR was performed using SYBR Green Fast qPCR Mix (ABclonal Technology) on a Bio-Rad CFX96 Real-Time System; Relative RNA abundance was calculated using the comparative Ct method (2^^-ΔΔCt^). The expression of Areg/P-Egfr/Egfr/Bcl-2/Caspase3/Cleaved Caspase-3 proteins was examined.

### *In vivo* imaging

4T1 tumor-bearing mice were intravenously injected with 20 mg·kg^-^¹ of PBR or PB labeled with IR783. The fluorescent signals in the mice were observed at 0, 1, 3, 8, and 24 h using an* in vivo* bioluminescence image system. The fluorescence intensity of each isolated organ was measured at 24 h after the administration of PBR or PB labeled with IR783 (20 mg·kg^-^¹). PBR was dissolved in aqueous solutions at varying concentrations (0, 12.5, 25, 50, and 100 μg·mL^-^¹) for *in vitro* PA imaging using a near-infrared (NIR) PA imaging system at 808 nm. The *in vivo* PA of PBR (20 mg·kg^-^¹) was assessed with nude mice bearing 4T1 tumors at different times.

### PBR-mediated radiosensitization *in vivo*

To evaluate the radiosensitization effects of PBR, 4T1 tumor-bearing mice were randomly divided into four groups: saline, IR, PBR, and IR + PBR groups (n=5). Based on different treatment protocols, mice were intravenously injected with the PBR (20 mg·kg^-^¹) on day 0. The mice were irradiated on days 2 and 6 when needed (4Gy). The mice's body weight and tumor size were recorded during the treatment. Tumor tissues were collected from the mice at the end of the treatment, embedded in paraffin, sectioned, and subjected to Tunnel immunofluorescence and Ki67 immunohistochemical staining for pathological analysis.

### PBR-mediated radiosensitization synergistic with PD-L1 blockade

A bilateral tumor model was established using female BALB/c mice to validate the therapeutic efficacy of PBR-mediated radiotherapy combined with PD-L1 antibodies. Initially, 4T1 cells (1×10^7^ mL^-1^, 100 μL) were injected into the subcutaneous tissue of the right leg, followed by an injection of cells (3×10^6^ mL^-1^, 100 μL) into the subcutaneous tissue of the left leg seven days later. The mice were randomly assigned to the following groups: (1) saline, (2) IR + anti-PD-L1, (3) PBR (20 mg·kg^-^¹) + anti-PD-L1, (4) PBR+IR+anti-PD-L1. According to the protocol, the groups underwent irradiation (4 Gy) or intraperitoneal injection of PD-L1 antibody (75 μg·kg^-^¹) on days 2, 5, and 8. The tumor size was measured and recorded every other day. The serum levels of the pro-inflammatory factors TNF-α, IFN-γ, and IL-6 were measured using ELISA kits.

### Dendritic cell maturation and T Cell infiltration

Maturation of dendritic cells (DC) and T-cell infiltration in mice were analyzed using flow cytometry. 4T1 tumor-bearing mice were intravenously administered with PBR at 20 mg·kg^-^¹, followed by 4 Gy irradiation at the tumor site after 24 h. Lymph nodes and spleens were collected 4 days after treatment. These tissues were homogenized in RPMI 1640 and washed with PBS after removing red blood cells. Single cells were obtained using a 70 μm cell strainer and dispersed in the flow cytometry staining buffer. The cell suspension was treated on ice for 30 min with 7-AAD, APC-Cy7 anti-mouse CD3, FITC anti-mouse CD4, and PE anti-mouse CD8a antibodies to investigate T-cell infiltration in the spleen. Lymph node cell suspensions were treated with 7-AAD, PE-conjugated anti-mouse CD86, APC-conjugated anti-mouse CD11c, and FITC-conjugated anti-mouse CD80 antibodies to assess DC maturation further.

### Statistical analyses

All data are expressed as the mean ± standard deviation (SD). All results were replicated in at least three independent experiments. Statistical analyses were performed using the GraphPad Prism 8 (GraphPad Software LLC, San Diego, CA, USA). For unpaired data, the Student's *t*-test was used for statistical comparisons. Statistical significance was set at *P* < 0.05.

## Supplementary Material

Supplementary materials and methods, figures and tables.
