# Supplementary material for: Bioeliminable Pt@Bi2Se3-RGD Nanoassembly for Enhancing Photoacoustic Imaging-Guided Tumor Immuno-Radiotherapy by Inducing Apoptosis via the Areg Pathway
Source: Theranostics. 2025 Jan 27;15(7):2720–36. doi: 10.7150/thno.106479 (PMC11898289; doi:10.7150/thno.106479)
Supplement: Supplementary file 1 — Supplementary materials and methods, figures and tables. [file thnov15p2720s1.pdf]

Bioeliminable Pt@Bi_2Se_3-RGD Nanoassembly for Enhancing Photoacoustic Imaging-Guided Tumor Immuno-Radiotherapy by Inducing Apoptosis via Areg Pathway

Huanhuan Tan[#1], Shiyan Fu[#1], Li Shen[1], Qinyang Lin[1], Wenrun Li[1], Yonghong Ran[1], Yazhen Zhao[1], Longfei Tan[2], Yuhui Hao*[1]

1. State Key Laboratory of Trauma and Chemical Poisoning, Institute of Combined Injury, Chongqing Engineering Research Center for Nanomedicine, College of Preventive Medicine, Army Medical University, Chongqing 400038, China

2. Laboratory of Controllable Preparation and Application of Nanomaterials, Key Laboratory of Cryogenics, Technical Institute of Physics and Chemistry, Chinese Academy of Sciences, 29 Zhongguancun East Road, Beijing 100190, PR China

*Corresponding authors: Yuhui Hao (Email: yuhuihao@tmmu.edu.cn).

[#]They contributed the same to this article.

Materials

Bismuth nitrate pentahydrate ($Bi(NO_3)_3 \cdot 5H_2O$), sodium hydroxide (NaOH), polyvinylpyrrolidone (PVP)， ethylene glycol (EG), silk sericin (SS), ascorbic acid (AA), sodium selenite (Na_2SeO_3), chloroplatinic acid (H_2PtCl_3), sodium borohydride ($NaBH_4$), 1-(3-dimethylaminopropyl)-3-ethylcarbodiimide (EDC), N-hydroxysuccinimide (NHS), Crgd-PEG-amine (cRGD-PEG-NH_2), dimethyl sulfoxide (DMSO), terephthalic acid (TPA) and hydrogen peroxide (H_2O_2, 30%) were bought from Shanghai KeShi Technology Co. Ltd. (China). Nystatin and Me-β-CD were bought from MACKLIN (China). Amiloride was bought from Aladdin (China). CPZ was bought from Sigma. Dulbecco's modified Eagle's medium (DMEM), a penicillin (10000 $U \cdot mL^{-1}$)/streptomycin (10000 $\mu g \cdot mL^{-1}$) mixture, fetal bovine serum (FBS), and TrypLE™ express enzyme were acquired from Thermo Fisher Scientific (Waltham, MA, USA). FITC and IR783 were obtained from Sigma-Aldrich (St. Louis, MO, USA). Calcein/PI Cell Activity and Cytotoxicity Assay Kit, Lyso-Tracker Red (Lysosomal Red Fluorescent Probe), 2',7'-dichlorofluorescein diacetate (DCFH-DA), TUNEL apoptosis assay kit, phosphate-buffered saline (1×PBS), enhanced cell counting kit-8 (CCK-8), DAB horseradish peroxidase color development kit, Ki67 rabbit polyclonal antibody, phospho-EGFR (Ser695) rabbit monoclonal antibody, HRP-labeled goat anti-rat IgG(H+ L), HRP-labeled goat anti-rabbit IgG(H+L), Alexa Fluor 488-labeled goat anti-rabbit IgG(H+L), and Alexa Fluor 647-labeled goat anti-mouse IgG(H+L) were purchased from Beyotime Biotechnology (China). All the cell lines were obtained from the Type Culture Collection of the Chinese Academy of Sciences (Shanghai, China). Female BALB/c mice (8 weeks old, ~20 g) were from Hunan SJA Laboratory Animal Co. And maintained under controlled conditions of temperature (22-24 ° C) and humidity (40-60 %) with free access to water and standard feed. 7-AAD Viability

Staining Solution, FITC anti-mouse CD80, PE anti-mouse CD86, APC anti-mouse CD11c, PE anti-mouse CD8a, FITC anti-mouse CD4, and APC-Cy7 anti-mouse CD3 antibodies were purchased from BioLegend. Mouse TNF-α, IFN-γ, and IL-6 were part of ELISA kits obtained from mlbio (China). Anti-HMGB1, anti-CRT, anti-γH2AX, anti-Areg, anti-Phosopho-Egfr (Anti-P-Egfr, Tyr1068), anti-Egfr, anti-Bcl-2, anti-Caspase3, and anti-β-actin were acquired from Proteintech (Wuhan, China). Milli-Q water was prepared using a water purification system (Millipore, Burlington, MA, USA) and used in all the tests. All mouse experimental protocols were approved by the Laboratory Animal Ethics Committee.

Characterization

TEM images of the PBR nanoassembly and high-angle annular dark-field scanning transmission electron microscopy images were obtained using a FEI TALOS G2 high-resolution transmission electron microscope operating at 200 kV. The ζ-potential and size of the PBR were measured using a Malvern Zetasizer NANO ZS90. FTIR spectroscopy characteristics were recorded using a Thermo Fisher Scientific Nicolet iS20 FT-IR spectrometer. Elemental analysis was performed using an iCAP TQ system (Thermo Fisher Scientific). Moreover, X-ray photoelectron spectroscopy was performed using a Thermo Fisher Scientific ESCALAB 250Xi spectrometer. The crystalline morphology of the nanostructures was characterized through XRD using an X-ray diffractometer (XRD-7000; Shimadzu, Japan) with Cu K-α radiation (λ = 1.5406 Å). Ultraviolet-visible absorbance measurements were performed using a UV-3600 UV-visible spectrophotometer (Shimadzu). The X-ray parameters used were: 160 kVp and 25 mA, with a 0.3 mm copper filter. BS and B were analyzed by thermogravimetric analysis.

Supplementary data

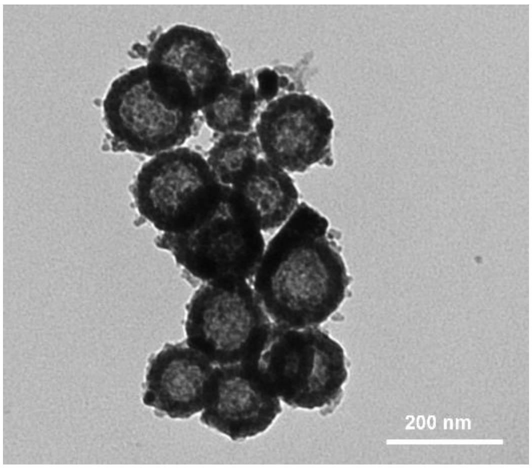

Figure S1. High-resolution transmission electron microscopy (TEM) image of Bi_2Se_3.

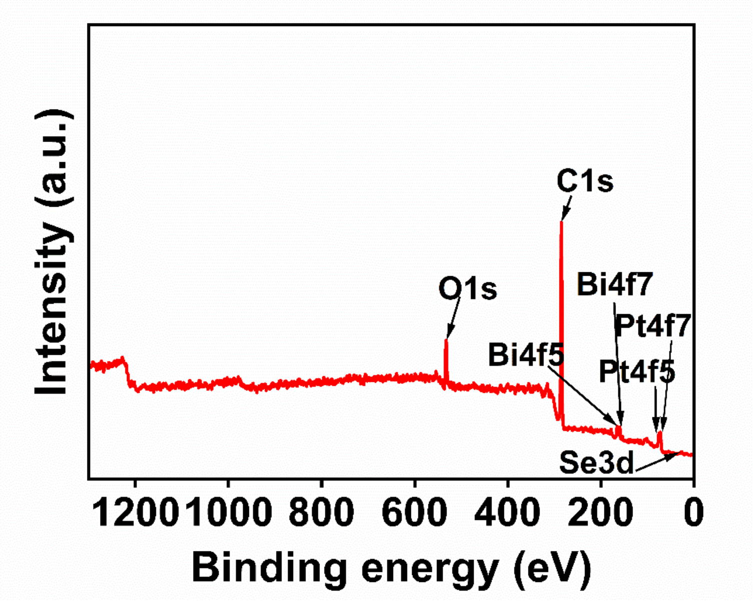

Figure S2. XPS pattern of full spectrum of PBR.

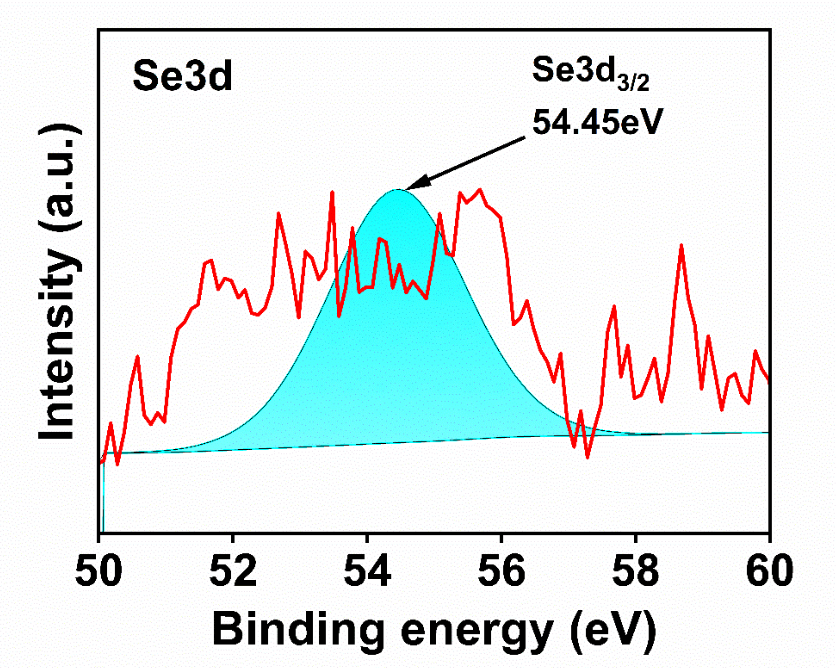

Figure S3. XPS spectrum spectra of Se3d in PBR.

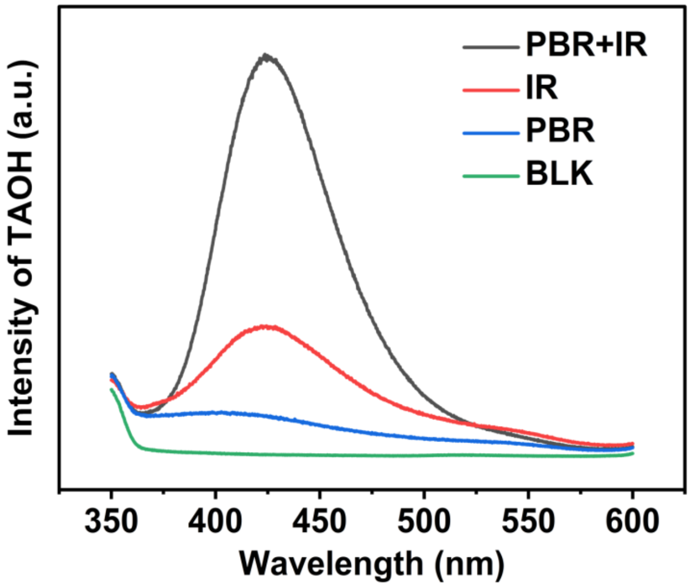

Figure S4. TPA as a tracing agent for •OH generated from PBR in the presence of irradiation (4 Gy).

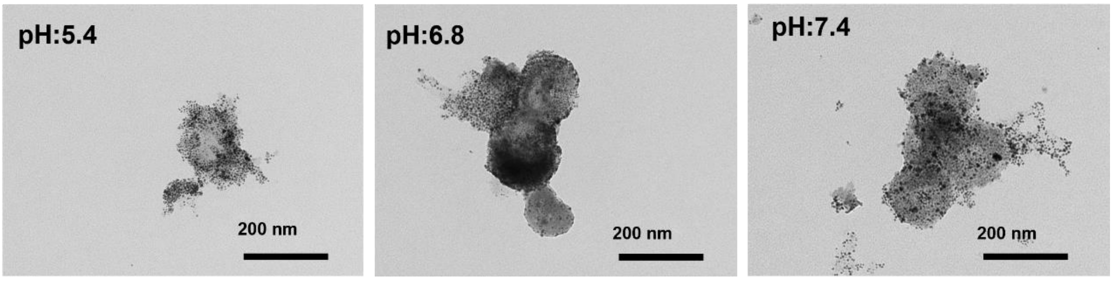

Figure S5. *In vitro* simulated biodegradation of PBR in Phosphate buffer solution of different pH (pH=5.4, 6.8, and 7.4) with Cathepsin B.

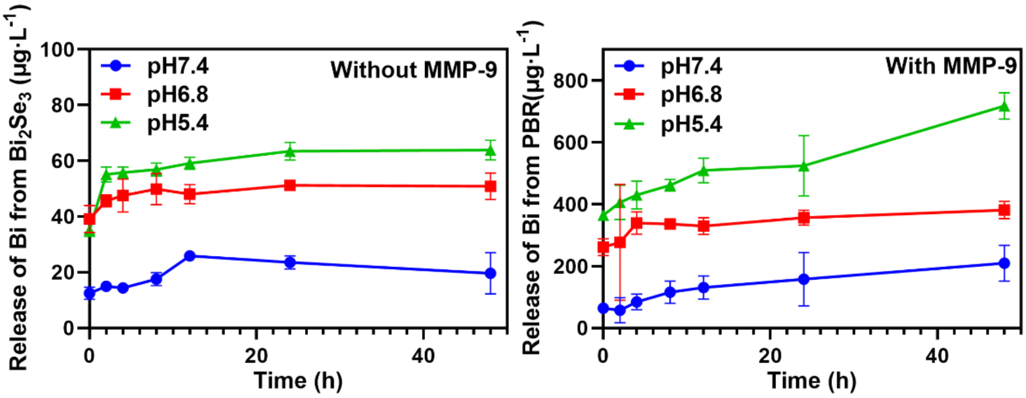

Figure S6. The release of Bi element from Bi_2Se_3 and PBR at different pH values detected by ICP-MS.

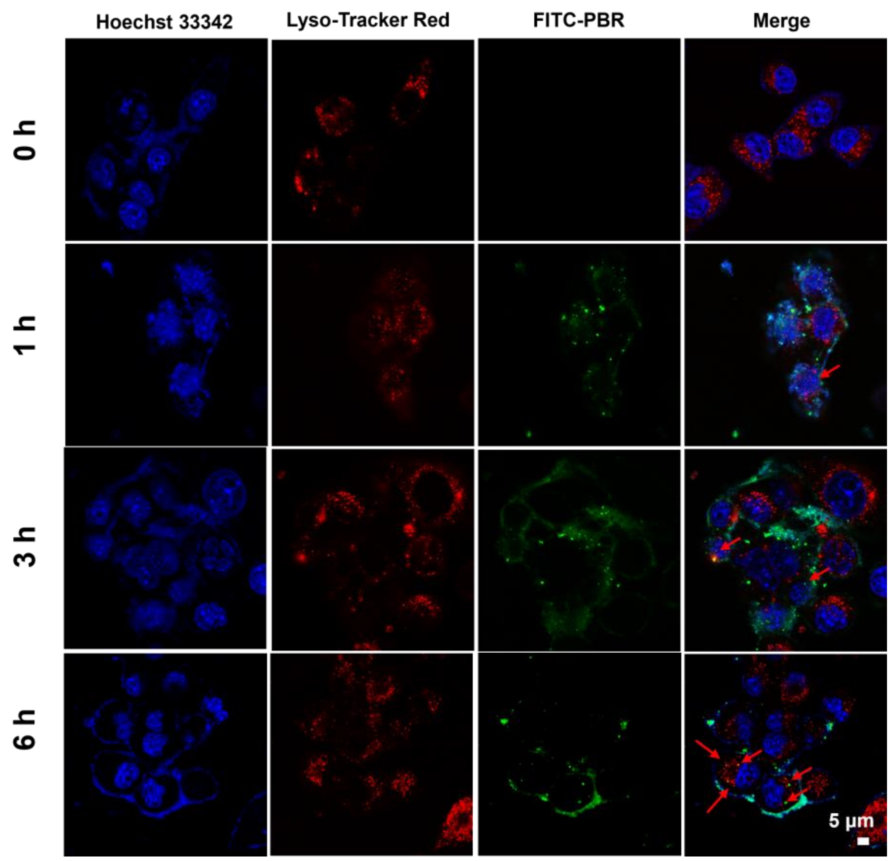

Figure S7. Confocal images showing the subcellular localization of PBR after various incubation time.

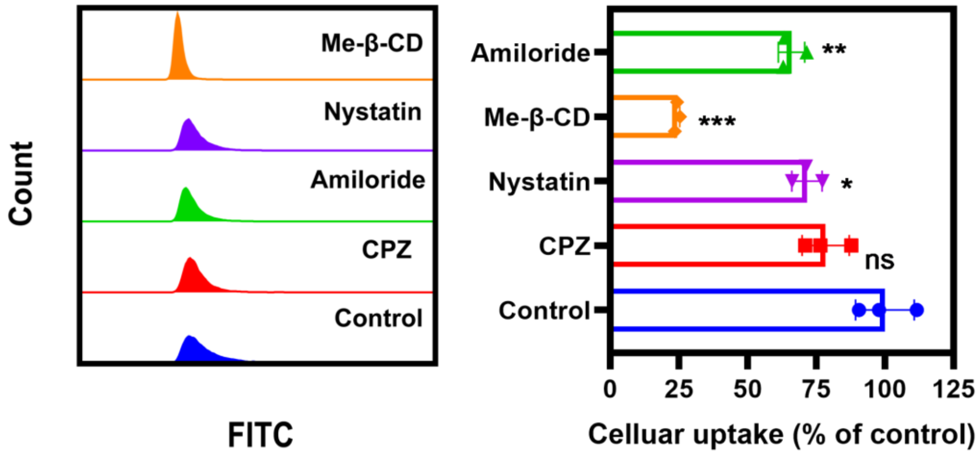

Figure S8. Flow cytometry analysis of cellular uptake of PBR in 4T1 cells after treated with different endocytosis inhibitors for 6h.

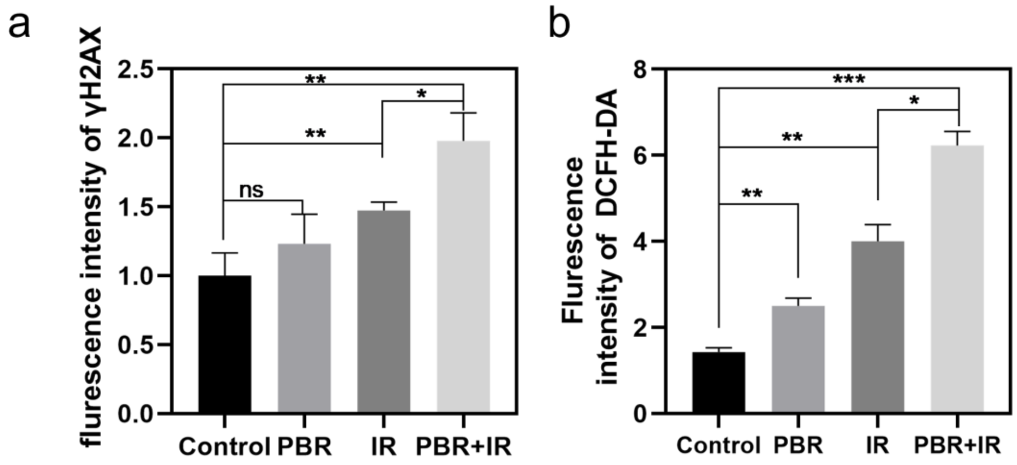

Figure S9 Fluorescence intensity of (a) γH2AX, (b) DCFH-DA.Statistical analysis was performed using a *t*-test. Data are presented as the mean ± SD. **P* < 0.05, ***P* < 0.01, ****P* < 0.001 and *****P* < 0.0001.

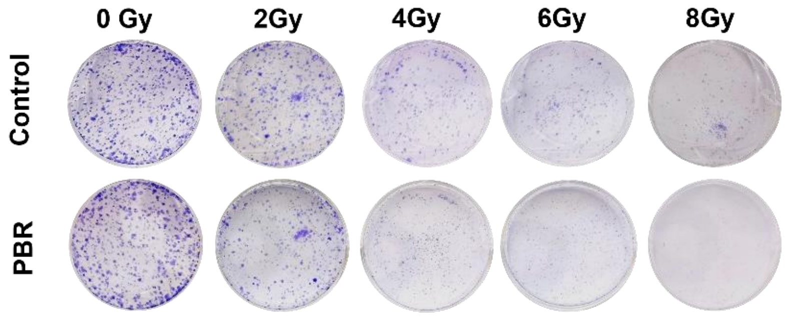

Figure S10. Colony formation of 4T1 cells incubated with or without PBR (25 $\mu g \cdot mL^{-1}$) and different irradiation doses(n = 5).

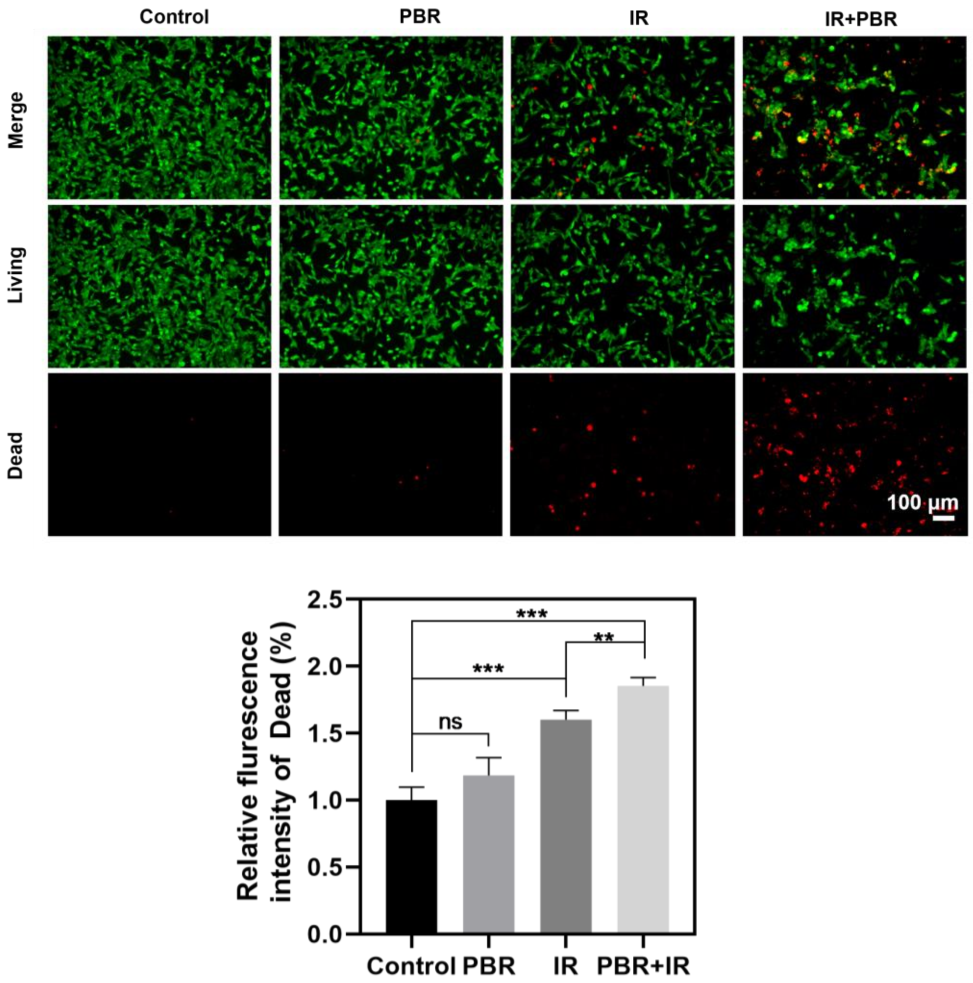

Figure S11. Cell death of 4T1 cells after PBR treatment with or without ionizing radiation (Ionizing radiation: 4 Gy, PBR incubation time: 48 h). Statistical analysis was performed using a *t*-test. Data are presented as the mean ± SD. *$P < 0.05$, **$P < 0.01$, ***$P < 0.001$.

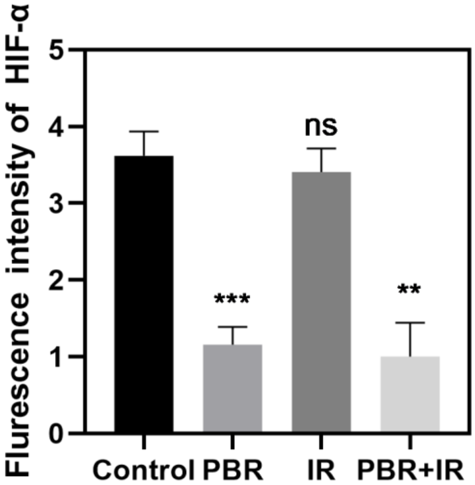

Figure S12. Fluorescence intensity of HIF-1α. Statistical analysis was performed using a *t*-test. Data are presented as the mean ± SD. **P* < 0.05, ***P* < 0.01, ****P* < 0.001.

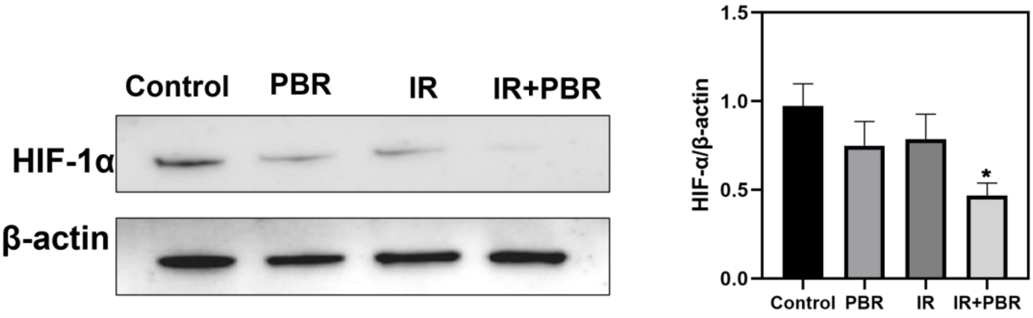

Figure S13. Western blot analysis and semi-quantitative analysis evaluating changes in the expression of HIF-1α in 4T1 cells following different treatments.Statistical analysis was performed using a *t*-test. Data are presented as the mean ± SD. **P* < 0.05, ***P* < 0.01, ****P* < 0.001.

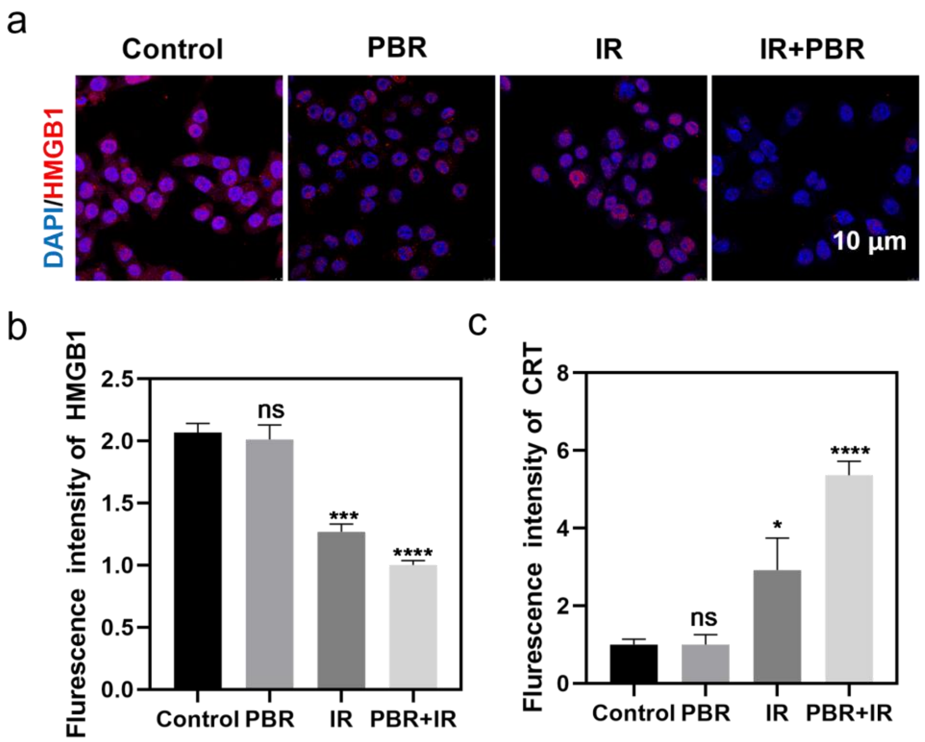

Figure S14. Fluorescence intensity of HGB1 (a-b) and CRT (c). Statistical analysis was performed using a *t*-test. Data are presented as the mean ± SD. *$P < 0.05$, **$P < 0.01$, ***$P < 0.001$ and ****$P < 0.0001$.

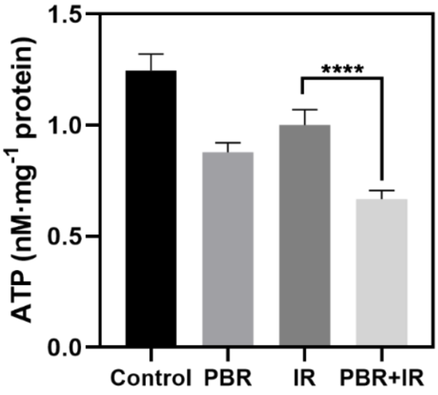

Figure S15. Intracellular ATP level changes after different treatments. Statistical analysis was performed using a *t*-test. Data are presented as the mean ± SD. *$P < 0.05$, **$P < 0.01$, ***$P < 0.001$ and ****$P < 0.0001$.

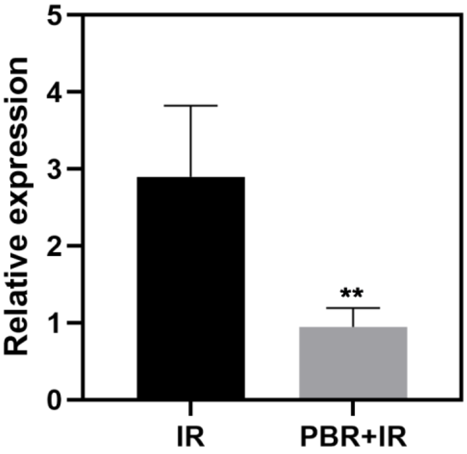

Figure S16. Transcription level of Areg was assessed by using real-time quantitative PCR (qPCR). Statistical analysis was performed using a *t*-test. Data are presented as the mean ± SD. *P < 0.05, **P < 0.01, ***P < 0.001.

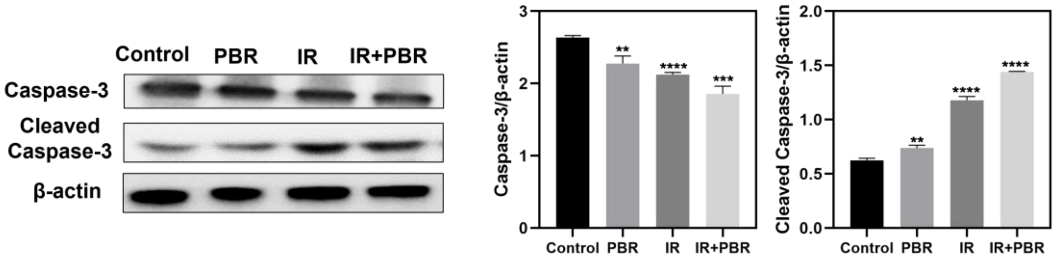

Figure S17. Western blot analysis and semi-quantitative analysis evaluating changes in the expression of Caspase-3 and Cleaved Caspase-3 in 4T1 cells following different treatments.Statistical analysis was performed using a *t*-test. Data are presented as the mean ± SD. *P < 0.05, **P < 0.01, ***P < 0.001 and ****P < 0.0001.

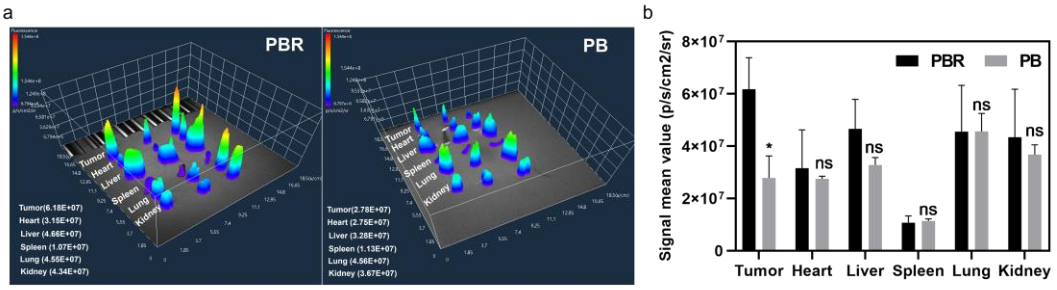

Figure S18. Fluorescence imaging of 4T1 tumor-bearing mice injected with IR783 labeled PBR or PB (20 mg·k^{-1}), and fluorescence distribution in excised organs and tumors 24 h after injection.Statistical analysis was performed using a *t*-test. Data are presented as the mean ± SD. **P* < 0.05, ***P* < 0.01, ****P* < 0.001.

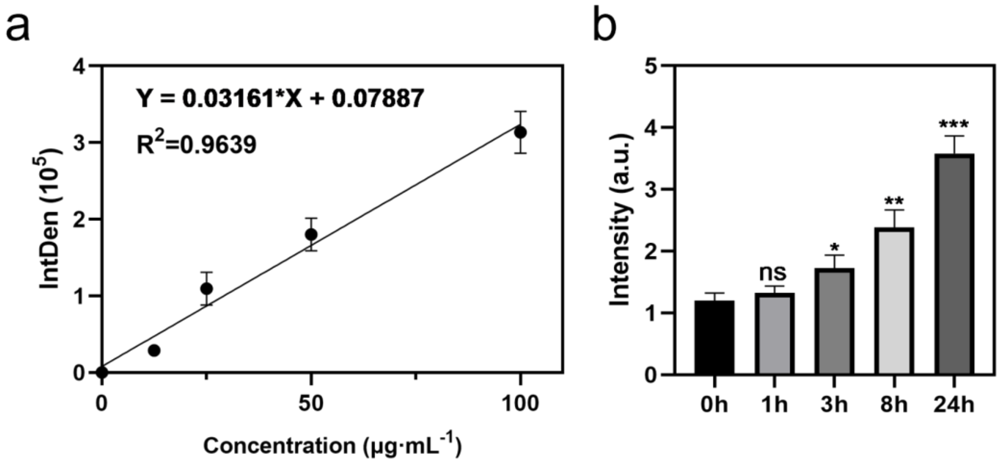

Figure S19. (a) The relationship between the photoacoustic imaging signal intensity and concentration of PBR. (b)Semi quantitative statistics of photoacoustic imaging signal intensity in tumor areas. Statistical analysis was performed using a *t*-test. Data are presented as the mean ± SD. **P* < 0.05, ***P* < 0.01, ****P* < 0.001.

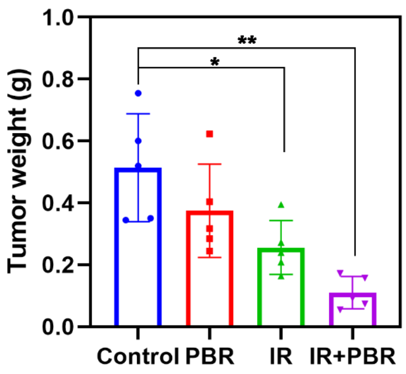

Figure S20. Weight of dissected solid tumors after two weeks post-treatment.Statistical analysis was performed using a *t*-test. Data are presented as the mean ± SD. **P* < 0.05, ***P* < 0.01, ****P* < 0.001.

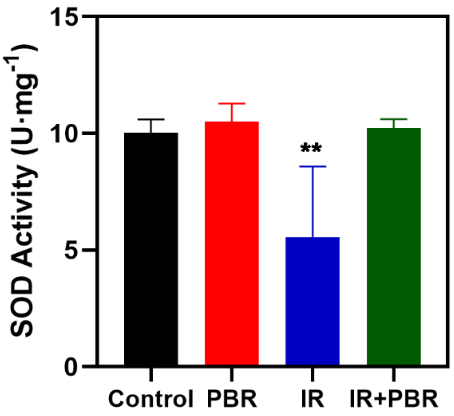

Figure S21. Serum levels of superoxide dismutase (SOD) activity in mouse after two weeks post-treatment.Statistical analysis was performed using a *t*-test. Data are presented as the mean ± SD. **P* < 0.05, ***P* < 0.01, ****P* < 0.001.

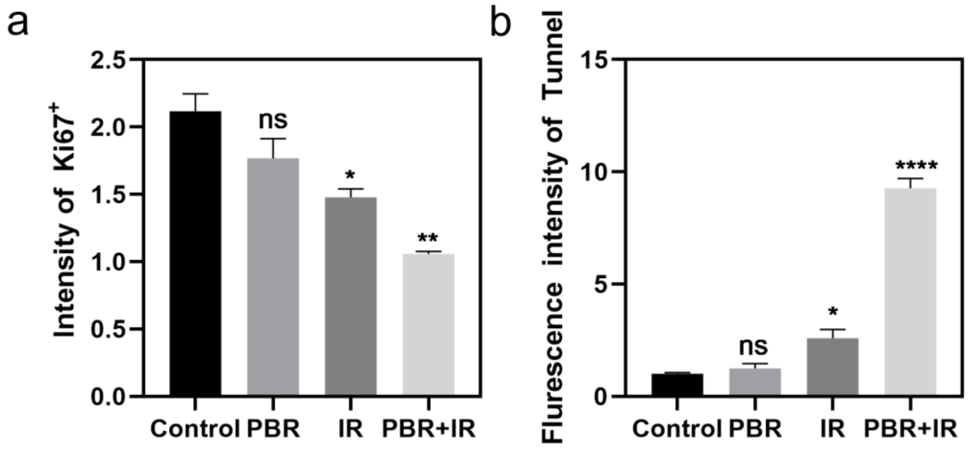

Figure S22. The Intensity of tumor sections by Ki67 immunohistochemistry (a) and Tunel immunofluorescence staining (b)Statistical analysis was performed using a *t*-test. Data are presented as the mean ± SD. $*P < 0.05$, $**P < 0.01$, $***P < 0.001$ and $****P < 0.0001$.

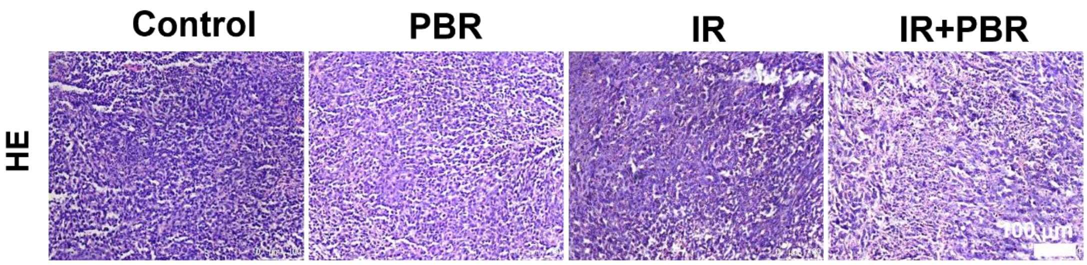

Figure S23. Histological analysis of tumor sections via Hematoxylin and Eosin (H&E) staining on day 14.

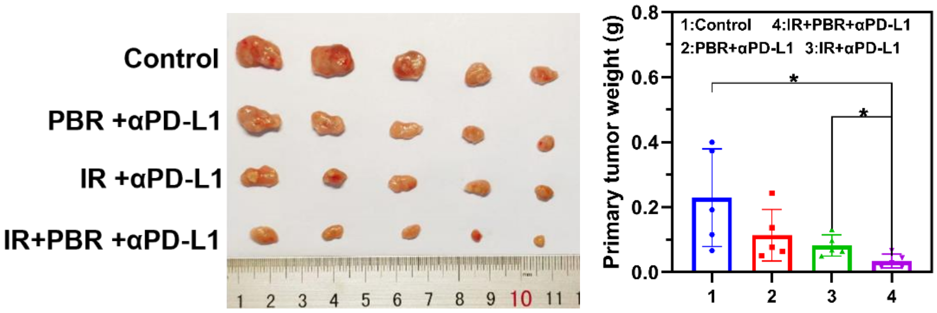

Figure S24. Graphs and weight of dissected primary solid tumor on day 14 after two weeks post-treatments.Statistical analysis was performed using a *t*-test. Data are presented as the mean ± SD. *$P < 0.05$, **$P < 0.01$, ***$P < 0.001$.

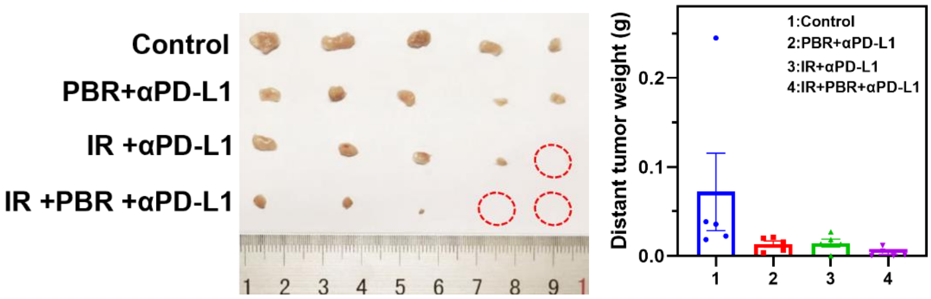

Figure S25. Graphs and weight of dissected distant solid tumor on day 14 after two weeks post-treatments. Statistical analysis was performed using a *t*-test. Data are presented as the mean ± SD. *$P < 0.05$, **$P < 0.01$, ***$P < 0.001$.

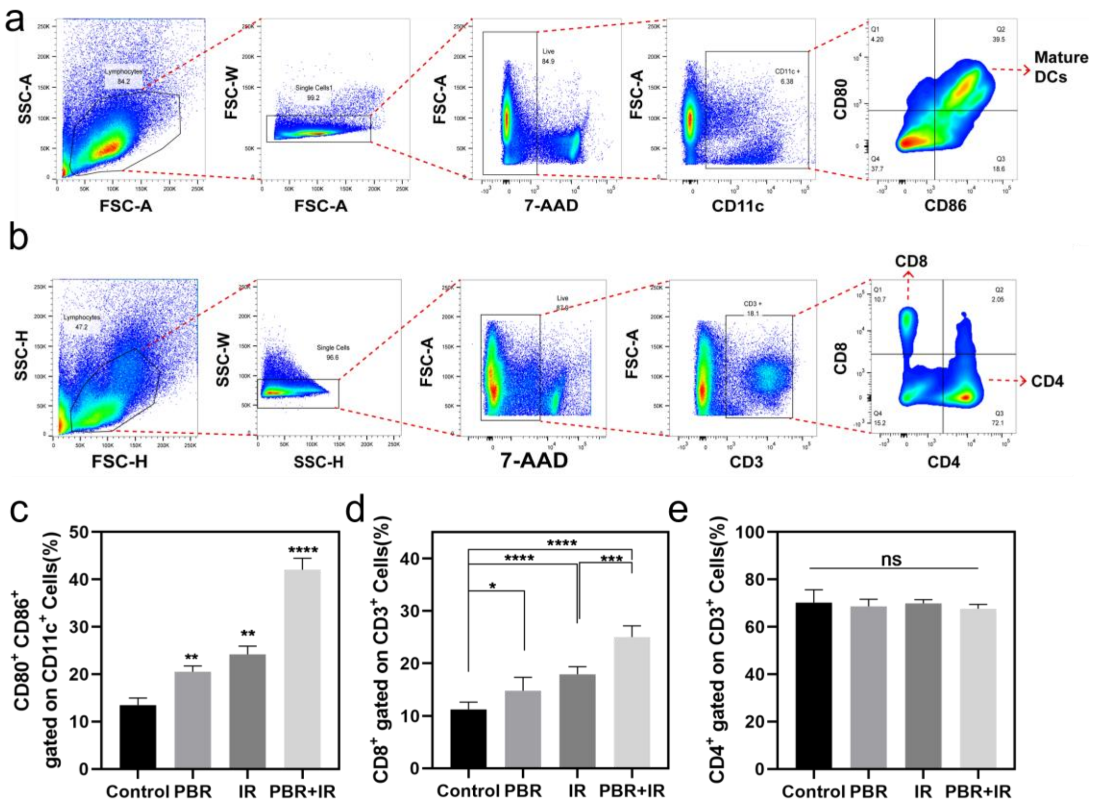

Figure S26. Representative FCM images of DC cells and T cells. (a)Gating strategies of CD80$^+$ and CD86$^+$ T cells in lymph tissues. (b)Gating strategies of CD3$^+$ and CD4$^+$ T cells in spleen tissues. CD80$^+$ and CD86$^+$ T cells of (c) lymph, and CD8$^+$ and CD4$^+$ T cells of (d-e) spleen tissues in the Control, PBR, IR, PBR +IR groups detected by FCM (n = 5 mice). Statistical analysis was performed using a *t*-test. Data are presented as the mean ± SD. *$P < 0.05$, **$P < 0.01$, ***$P < 0.001$ and ****$P < 0.0001$.

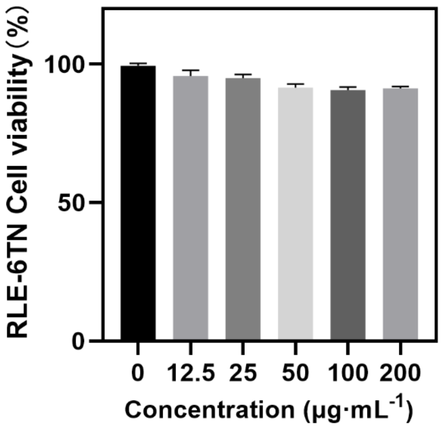

Figure S27. Viability of RLE-6TN cells after incubation with various concentrations of PBR for 48h.

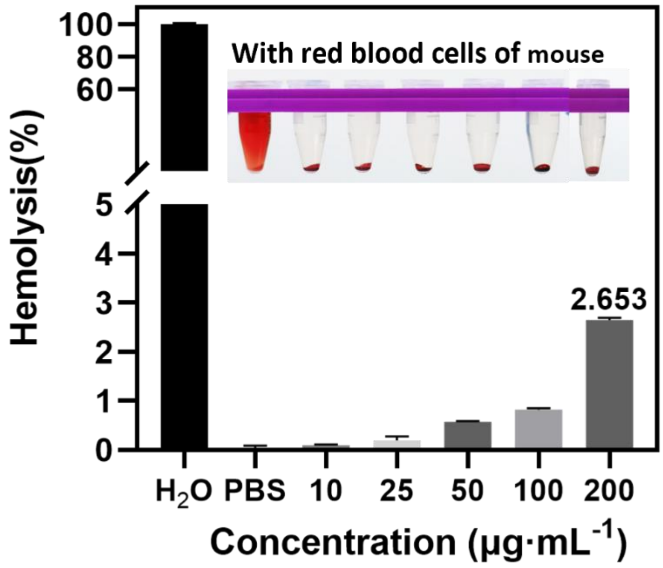

Figure S28. Hemolytic rate of erythrocytes after incubation with PBR at various concentrations at 37 °C for 3 h. DI water served as the positive control (100% of hemolysis rate). Inset: photograph of the centrifuge tubes containing various samples.

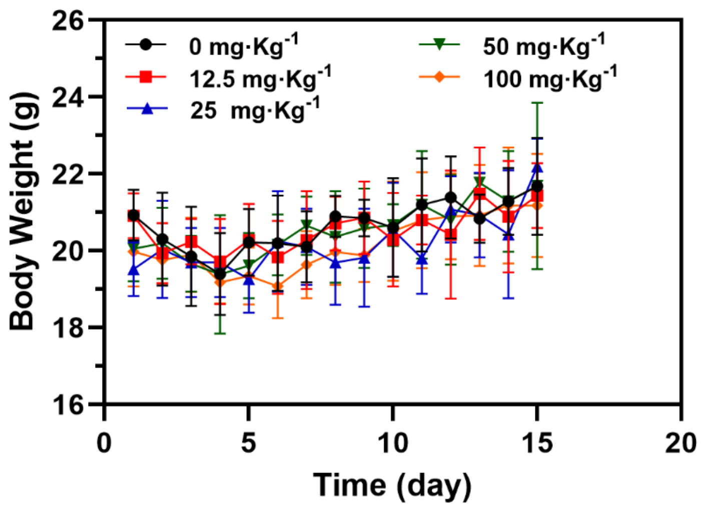

Figure S29. Variation of BALB/c mouse body weight after intravenous injection with PBR with different dosages during the treatment period of 15 days.

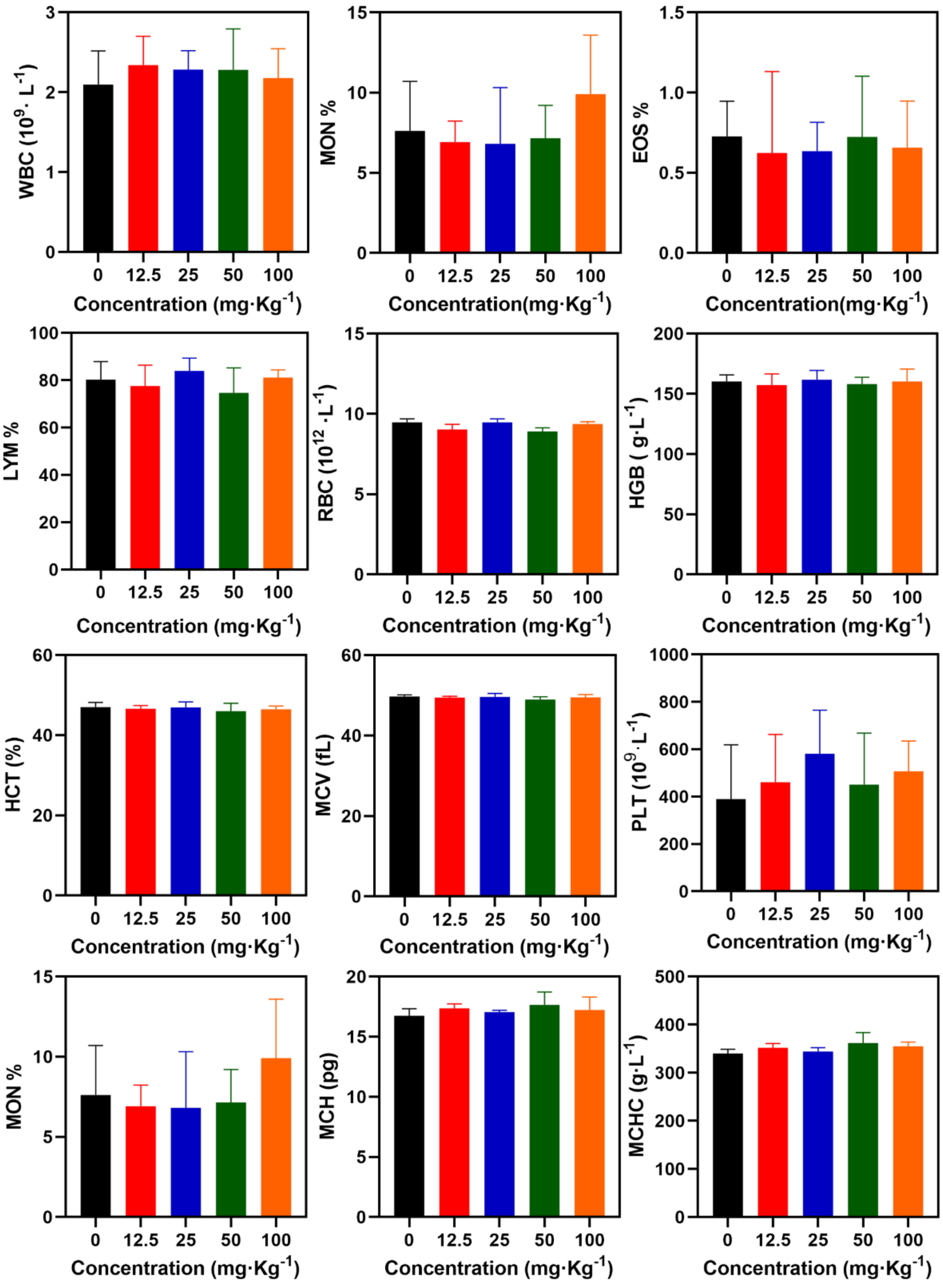

Figure S30. Primary indicators of blood routine test on day 15 after BALB/c mice being intravenously injected with PBR at different dosages.

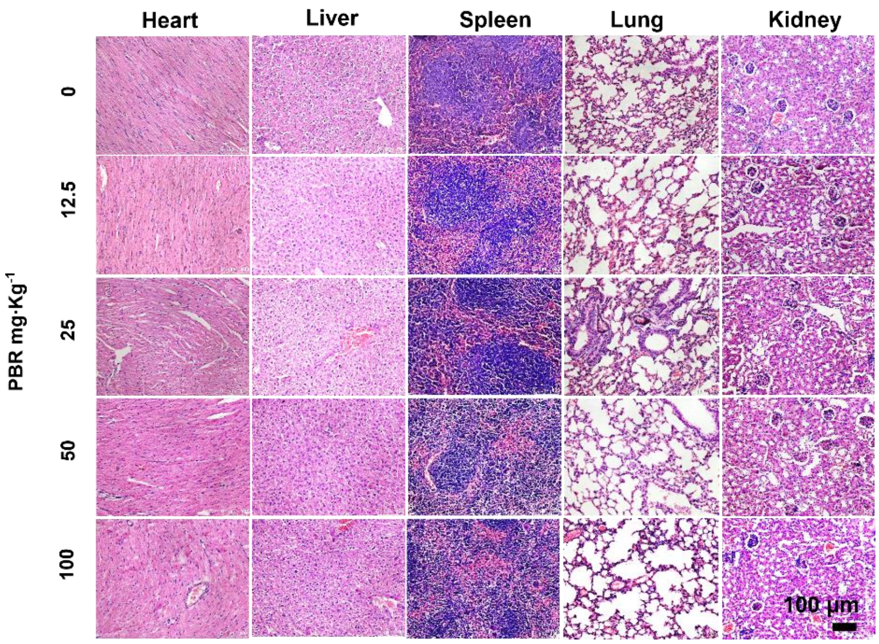

Figure S31. H&E staining of major organs (heart, liver, spleen, lung and kidney) harvested from BALB/c mice on day 15 after intravenous injection of PBR at different dosages.

Table S1. Differential gene statistics table

compare	all	up	down	shold
IR+PBR vs IR	1135	618	517	|log2FoldChange|>1&pvalue<0.05

Table S2. Reactome Pathway Enrichment Analysis Results Table (top minimum 5

padj PathWays)

Description	GeneRatio	BgRatio	pvalue	p.adjest	qvalue	geneNAME	Count
GRB2 events in EGFR signaling	4/76	12/8846	2.36E-06	0.000793	0.000719	Egf/Areg/Btc/Hbegf	4
SHC1 events in EGFR signaling	4/76	13/8846	3.39E-06	0.000793	0.000719	Egf/Areg/Btc/Hbegf	4
EGFR downregulation	5/76	30/8846	4.94E-06	0.000793	0.000719	Egf/Areg/Btc/Rps27a/Hbegf	5
GAB1 signalosome	4/76	17/8846	1.10E-05	0.001326	0.001201	Egf/Areg/Btc/Hbegf	4
Signaling by EGFR	5/76	45/8846	3.83E-05	0.003691	0.003345	Egf/Areg/Btc/Rps27a/Hbegf	5
